# Combinational Vibration Modes in H_2_O/HDO/D_2_O Mixtures Detected Thanks to the Superior Sensitivity of Femtosecond Stimulated Raman Scattering

**DOI:** 10.1021/acs.jpcb.3c01334

**Published:** 2023-05-18

**Authors:** Marcin Pastorczak, Katsiaryna Duk, Samaneh Shahab, Alexei A. Kananenka

**Affiliations:** † Institute of Physical Chemistry, Polish Academy of Sciences, Laser Centre, Kasprzaka 44/52, 01-224 Warsaw, Poland; ‡ Department of Physics and Astronomy, University of Delaware, Newark, Delaware 19716, United States

## Abstract

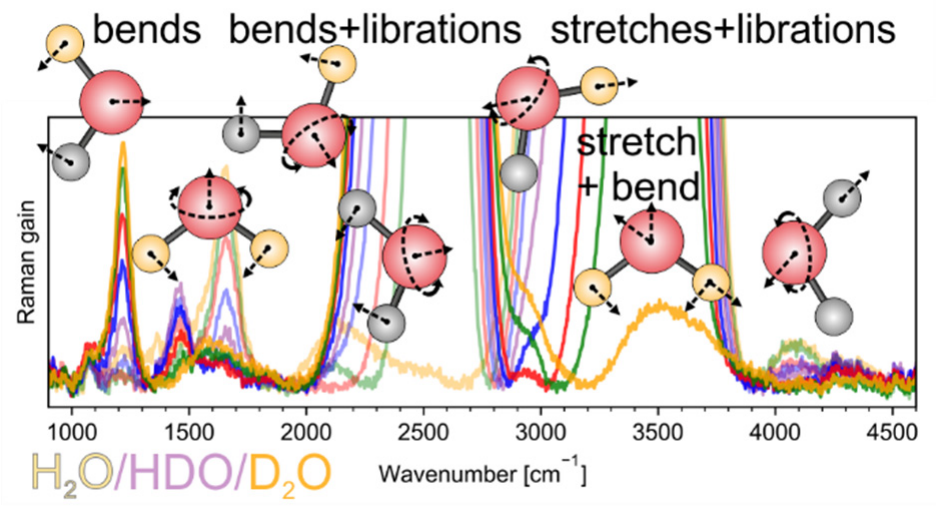

Overtones and combinational modes frequently play essential roles in ultrafast vibrational energy relaxation in liquid water. However, these modes are very weak and often overlap with fundamental modes, particularly in isotopologues mixtures. We measured VV and HV Raman spectra of H_2_O and D_2_O mixtures with femtosecond stimulated Raman scattering (FSRS) and compared the results with calculated spectra. Specifically, we observed the mode at around 1850 cm^–1^ and assigned it to H–O–D bend + rocking libration. Second, we found that the H–O–D bend overtone band and the OD stretch + rocking libration combination band contribute to the band located between 2850 and 3050 cm^–1^. Furthermore, we assigned the broad band located between 4000 and 4200 cm^–1^ to be composed of combinational modes of high-frequency OH stretching modes with predominantly twisting and rocking librations. These results should help in a proper interpretation of Raman spectra of aqueous systems as well as in the identification of vibrational relaxation pathways in isotopically diluted water.

## 1 Introduction

The isotopic dilution of liquid water (the substitution of H for D atom) results in significant changes in the vibrational spectrum of the system. The most striking change is the redshift of the stretching vibrations frequency from around 3400 cm^–1^ for the OH groups down to around 2500 cm^–1^ for the OD groups. In laboratory practice, such a dilution is often done to reduce normally colossal, water infrared absorption in the OH stretching region or to generate additional marker bands in the so-called “silent region” of a vibrational spectrum in biomedical spectroscopy.^1^ Another important effect of such dilution is the decoupling of OH or OD stretches from resonantly vibrating neighbors.^2−5^ This effect is often exploited in fundamental (usually time-resolved) spectroscopic studies of water vibrations.^5−7^ Moreover, a tiny fraction of vibrationally decoupled oscillators may be used to study the rotational diffusion of liquid water in various systems.^8,9^ Replacing H_2_O with heavy water also shifts the bending fundamental band from around 1645 cm^–1^^10^ to approximately 1200 cm^–1^ in D_2_O. This isotope shift is instrumental in infrared (IR) and two-dimensional infrared (2D IR) spectroscopy of proteins, where it allows for measurements of protein’s amide I vibrations (1620–1680 cm^–1^) free of solvent (H_2_O) interference.^116−118^

The isotopic substitution, let alone its usefulness, leads to the presence of the three HDO, D_2_O, and H_2_O isotopologues in a mixture, all having their vibrational manifestation in a spectrum. The fundamental modes of these components are well-known and frequently studied. In addition to already mentioned OD and OH stretching modes (symmetric and antisymmetric, strongly overlapped in the condensed phase) and H–O–H and D–O–D bending modes, there is also the H–O–D bending mode at around 1460 cm^–1^.^11^ Moreover, in the low frequency region, there are strongly overlapped librations, rotations of water molecules that are essentially hindered (restrained) by hydrogen bonds. There are three rotations that a “free” water molecule would have in the gas phase: around its *C*_2_ symmetry axis, in-plane, and out-of-plane. Correspondingly twisting librations around 450 cm^–1^ (*v*_L1_) are associated with hindered rotations of water molecules around the *C*_2_ symmetry axis, rocking librations around 550 cm^–1^ are hindered in-plane rotations (*v*_L2_), and wagging librations around 725 cm^–1^^12−15^ are restrained out-of-plane vibrations of water molecules (*v*_L3_). These bands are red-shifted by a factor of ≈√2 for D_2_O to, respectively, around 330, 425, and 565 cm^–1^.^15^ The so-called translational region 20–300 cm^–1^ contains two broad bands, signatures of low-frequency modes: hydrogen-bond bending at 62 cm^–1^ (*v*_T2_)^13,16,17^ and hydrogen-bond stretching at 175 cm^–1^ (*v*_T1_).^12,16,18−20^ These bands were also interpreted in terms of restricted translations of water molecules.^21,22^

Overtones and combinations of fundamental modes, due to very low intensity, are typically difficult to observe. Nevertheless, in the spectrum of the H_2_O/HDO/D_2_O mixture, one should expect overtones and combinational modes originating from all three compounds. Walrafen and co-workers predicted the spectral positions of most of the combination bands and observed some.^15,23^ However, the then-existing state of technology allowed for determination of the position of these “weak bands” just from the “negative/positive concavities” of a baseline.^15^ Recently, Verma et al. pointed at the intermolecular nature of the combinational band bend + libration of H_2_O at 2130 cm^–1^, designating it as an excellent probe of water-hydration behavior.^24^ Therefore, this band is also called the “association band”.^25,26^ Also recently, the discussion on the nature of the band around 4100 cm^–1^ has been reignited. While Walrafen and Pugh assigned the band to the combination of low frequency part of the OH stretching mode with twisting libration,^23^ Morawietz et al. correlated the band with the combination of the high frequency part of the OH stretch and water wagging librations.^27^

In this work, we studied H_2_O/HDO/D_2_O mixtures with different concentrations of isotopologues with polarization-resolved femtosecond stimulated Raman scattering spectroscopy (FSRS).^28,29^ This method offers numerous advantages over spontaneous Raman scattering also in stationary (not time-resolved) studies. While only one Raman photon out of a million pump photons is produced in spontaneous methods, in stimulated Raman, that efficiency may be up to 10%.^30^ Moreover, the fluorescence background is suppressed in FSRS, and the transmission geometry of the measurement with the whole SRS signal contained in the coherent Raman probe beam allows for very high reproducibility of the experimental condition from sample to sample. Moreover, measurements for two relative polarizations (perpendicular and parallel) between the Raman pump and probe offer additional information on the symmetry of observed modes. In perpendicular polarization spectra, the intense symmetric components are not present, which facilitates observation of very weak asymmetric modes. Our highly sensitive FSRS studies revealed, for the first time, some combinational modes and provided additional features of the known modes.

Simulations have been integral in interpreting experimental spectra of liquid water.^27,31−,47^. In this work two computational approaches were employed. Mixed quantum-classical line shape theory based on electrostatic spectroscopic maps^31,32^ was used to calculate Raman spectra of H_2_O/D_2_O mixtures. Within this approach, low-frequency vibrational modes are treated classically via molecular dynamics (MD) simulations, whereas the high-frequency vibrations are treated quantum-mechanically. Specifically, the OH(OD) fundamental stretching modes and H–O–H(D–O–D/H–O–D) bending overtone modes are treated based on the exciton Hamiltonian expressed in the local mode basis. This approach has been used to calculate IR, 2D IR, and Raman spectra of liquid water and ice and is known to provide a good agreement with experiments.^32,35,44,48^

To interpret the origin of combination bands formed between high- and low-frequency vibrations, Raman spectra of the three isotopologues of the book isomer of the water hexamer were studied using vibrational perturbation theory (VPT2).^50^ The anharmonic treatment of vibrations is necessary because, as shown previously,^25^^51^ the description of combination bands of water in terms of harmonic normal modes is inadequate. Calculated combination bands allowed us to identify the dominant modes forming combination bands in terms of low- and high-frequency modes.

## 2 Experimental Section

### 2.1 Sample Preparation

The H_2_O/HDO/D_2_O mixtures of different compositions were prepared by mixing ultrapure H_2_O (from Millipore Direct Q3 UV system) with D_2_O (Cambridge Isotope Laboratories Inc., 99.9% D atoms) with the following initial (not equilibrated) concentrations of H_2_O: 0, 1, 5, 15, 30, 50, 70, 85, 95, and 100 mol %. The solutions were subsequently left in sealed glass flasks for equilibration for at least 1 day.

### 2.2 Spectroscopic Measurements

The stimulated Raman scattering studies were performed with the setup for pump–probe femtosecond stimulated Raman scattering, which was described in refs (52 and 53), but only the stationary state measurements were done here. The setup is based on a femtosecond Yb:KGW laser system (Pharos, Light Conversion) that produces 200 fs pulses centered at around 1030 nm with a repetition rate of 1 kHz. The setup is equipped with beam direction stabilization systems. A Raman pump was generated in a home-built picosecond OPA (optical parametric amplifier) in the process of frequency mixing of two oppositely chirped copies of the same femtosecond pulse (that method and the construction of OPA are described in refs (54 and 55)). The thus generated Raman 515 nm pump has a narrow bandwidth (∼5 cm^–1^) and is around 3 ps long. The Raman pump energy was around 0.5 μJ. The beam waists of the pumps in the focal points in a sample were about 25 μm (1/*e*^2^). The femtosecond Raman probe beam (supercontinuum) was generated by focusing a small portion of the laser pulse on a sapphire plate. The measurements were performed with both parallel (VV) and perpendicular polarization (HV) between the Raman pump and Raman probe in forward (transmission) geometry. Samples were measured in a 2 mm sample-path fused silica cuvette (Hellma QS quartz) at 20 °C. The cuvette was placed in a cuvette holder with a fixed position, and only the cuvette content was changed, which assured a fixed geometry (beams position on a sample, beams space and time overlap) between measurements. The Raman pump and the probe were temporarily overlapped with the use of a manual optical delay line. The Raman probe spectrum with and without the presence of the Raman beam (a chopper, Thorlabs, blocks every second Raman pump pulse) in the sample was recorded by the spectrometer (spectrograph Andor Shamrock SR 500i with CCD camera Andor Newton U971N).

### 2.3 Data Processing

The baseline (polynomial) was subtracted from each spectrum with the nod points of the polynomial attached at the same wavenumbers of a spectrum. Spectra were not normalized. For each composition of the H_2_O/D_2_O mixture, the equilibrium concentrations for components (H_2_O, D_2_O, and HDO) were calculated with the use of the following equation:
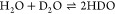

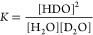
1with the equilibrium constant *K* ≈ 3.9.^56^ The spectra referred to as the “HDO spectra” below were extracted from the spectra of H_2_O/D_2_O mixtures by subtracting spectra of pure components (H_2_O, D_2_O) multiplied by their respected equilibrium fractions (*f*_H2O_, *f*_D2O_) from the spectra of the mixtures calculated by solving eq 1.

## 3 Computational Details

Mixed quantum-classical line shape theory was used to calculate VV and HV Raman spectra of pure H_2_O (D_2_O), as well as H_2_O/D_2_O/HDO mixtures. For a detailed description of the approach, the reader is referred to refs (32, 35, and 57) and Supporting Information. It was developed for the spectroscopy of molecular condensed-phase systems with coupled vibrational modes (chromophores). The main idea is to describe low-frequency vibrations using classical MD simulations and then treat the chosen high-frequency vibrations quantum-mechanically. This approach accounts for the inhomogeneous broadening and motional narrowing effects. In the quantum-mechanical treatment, the system of coupled vibrational chromophores is described by the excitonic Hamiltonian expressed in the local mode basis. The excitonic Hamiltonian is constructed by including the vibrations of interest. In this work, OH and OD stretching bands were modeled by the excitonic Hamiltonian with the diagonal elements being the OH/OD hydroxyl stretching and H–O–H, H–O–D, and D–O–D bending overtone local-mode frequencies as done previously.^35,48^ By including the bending overtone chromophores, we account for the stretch–bend Fermi resonance, which is believed to contribute to the OH/OD stretching band.^35,58−65^ The off-diagonal elements of the excitonic Hamiltonian are the vibrational couplings. There are two types of couplings between hydroxyl stretching chromophores: intramolecular and intermolecular. The former arises between two hydroxyl stretching modes of the same water molecule, while the latter corresponds to two hydroxyl stretching modes that belong to two different water molecules. Bending overtone chromophores are intramolecularly coupled to hydroxyl stretch chromophores via Fermi coupling.^35^

The diagonal frequencies and intramolecular OH(OD) couplings were modeled by the electrostatic maps^31,67^ that correlate the local mode frequency with the electric field created by the point charges of all atoms comprising the local atomistic environment of the water molecule. The charges were taken directly from the water model used in MD simulations described below. Intermolecular couplings between OH(OD) stretch chromophores were calculated within the transition dipole approximation.^32,68^ In the absence of the electrostatic map for the H–O–D bending overtone, the corresponding local-mode frequency was assumed to be independent of the environment and set at its experimental value of 2950 cm^–1^.^59^ How strongly the H–O–D bending overtone frequency depends on the atomistic environment of the HDO molecule is still an open question. The approximation used in this work is expected to work well, because the frequency of the H–O–D bending overtone remains essentially unchanged from that of its value fixed in the simulations due to a frequency mismatch with other high-frequency vibrations that were treated quantum-mechanically in this work. Similarly, the local mode frequency of D–O–D bending overtone was assumed independent of the environment and set to its experimental value of 2380 cm^–1^.^69^ This assumption is more questionable, although the dependence of D–O–D bending overtone frequency on the atomistic environment of the D_2_O molecule is unclear. It is known that including an electric-field-dependent H–O–H bending overtone map in the calculation of the OH stretching band does improve the agreement with the experiment.^35^ However, the effects of stretch–bend Fermi resonance on the OD stretching bands have not been computationally studied. In the future, it would be highly desirable to parametrize H–O–D and D–O–D fundamental and overtone bending maps as it has been done for the H–O–H bending fundamental and overtone vibrations.^35^^70^

Another necessary quantity for calculating Raman spectra, transition polarizability of the OH(OD) stretch chromophores is approximated using the bond polarizability model.^71^ The ratio between longitudinal and transverse bond polarizability derivatives is taken to be 5.6.^72^ More details can be found in refs (35 and 48) and in the Supporting Information. Because of the negligible intrinsic oscillator strength of the bending overtones, the corresponding transition polarizability was set to zero. It should be noted that bending overtone chromophores still contribute to the spectrum due to intensity borrowing from the fundamental OH(OD) chromophores via the intramolecular Fermi coupling.

Diagonal frequencies, excitonic couplings, and transition polarizabilities change in time because a fluctuating local environment gives rise to changing electric fields. These effects are captured by spectroscopic maps. The calculation of Raman spectra amounts to generating the excitonic Hamiltonian and the transition polarizability tensor for each MD configuration and computing the corresponding VV and HV response functions.^73^ The intrinsic lifetime broadening is accounted for approximately based on the experimentally measured lifetime of the vibrational excited states.

Our classical MD simulations employed a nonpolarizable three-body water model E3B2.^74^ Raman spectra of pure liquid water calculated using the mixed quantum-classical approach described above with E3B2 water model have shown to be in very good agreement with experiment.^35^ As compared in ref (61), such an approach yields Raman spectra that are in better agreement with experiment than *ab initio* MD simulation based on density functional theory and many-body water models. Additionally, we performed calculations with other popular water models: E3B3,^75^ TIP4P,^76^ TIP4P/2005,^77^ and SPC/E.^78^ We verified that VV and HV Raman spectra computed with the E3B2 water model are indeed in the best agreement with the experiment, although other models produced Raman spectra that are close to those of the E3B2 model.

MD simulations were performed in the NVT ensemble with 500 H_2_O molecules using GROMACS package version 4.5.5^65,79^ modified to implement an E3B2 water model. The simulation box size was scaled to reproduce the experimental density of liquid H_2_O at 298 K. Three-dimensional periodic boundary conditions were applied. Electrostatic interactions were calculated using the particle-mesh Ewald summation.^80^ The cutoff for Lennard-Jones interactions was set to 0.95 nm. The Nose-Hoover algorithm^81,82^ with a 2 ps coupling constant was used to maintain the system at the constant temperature of 298 K. The classical equations of motion were integrated with a 1 fs time step using the SETTLE algorithm.^83^ After an equilibration run of 1.0 ns, a production run of 2.0 ns was performed. During the production run, the atomic coordinates were saved every 10 fs for spectral calculations. The lifetime of vibrational excited states is taken to be 260 fs.^84^

Raman spectra of pure H_2_O were calculated directly based on the MD trajectory generated as described above. Raman spectra of pure D_2_O as well as of H_2_O/HDO/D_2_O mixtures were obtained as follows. Starting with the MD trajectory, the number of molecules in the H_2_O/D_2_O/HDO mixture was determined by solving eq 1. After that, the appropriate number of H_2_O molecules from the MD trajectory was designated to be HDO and D_2_O molecules, which are chosen randomly. The composition of the H_2_O/HDO/D_2_O mixture remained constant during the spectroscopic calculations. The VV and HV Raman spectra were calculated as explained in ref (35) and in the Supporting Information. The MultiSpec package was used for calculating all spectra. The MultiSpec package and example input files for calculating VV and HV Raman spectra from GROMACS trajectories can be downloaded at https://github.com/kananenka-group/MultiSpec.

**Figure 1:**
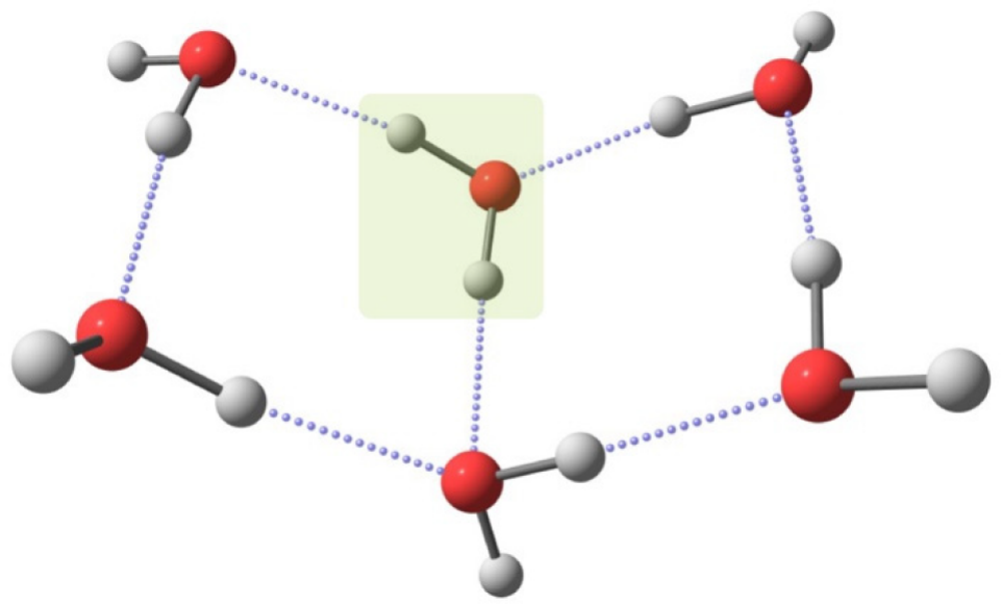
Book isomer of the water hexamer. The H_2_O molecule that was replaced by HDO and D_2_O molecules in VPT2 calculations is highlighted.

The mixed quantum-classical approach described above can be used to calculate high-frequency vibrational bands for which the local mode description is clear. The highly collective nature of librational modes and combination bands formed by these modes and high-frequency vibrations render the application quantum-classical approach difficult in these cases. To gain insight into the combination bands arising in the Raman spectra of liquid water, we turned our attention to water clusters. Specifically, we focused on the water hexamer. The water hexamer is the smallest water cluster that does not have a ring topology. It is often studied as a prototypical system to understand the molecular structure, dynamics, and spectroscopy^35,85,86^ of water^87^.^88^ Among the three most stable hexamer conformers^85,89–93^, we focus on the book isomer shown in Figure 1.

As shown by McCoy, the harmonic analysis based on normal modes cannot be used to study combination bands.^25,51^ Therefore, to study the progression of combination bands in the water hexamer upon isotope substitution, anharmonic frequencies, Raman activities, and depolarization ratios^94^ for the three hexamer clusters (H_2_O)_6_, (H_2_O)_5_D_2_O, and (H_2_O)_5_(HDO) were calculated using vibrational perturbation theory (VPT2),^50^ which is a widely used method for the anharmonic analysis of molecular vibrations of water.^95^ VPT2 calculations were performed using density functional theory with the B3LYP^96,97^ functional and 6-311++G(d,p)^98^ basis set. All calculations were performed using the Gaussian 16^99^ software package. Before calculating anharmonic frequencies, the geometry of the book hexamer was optimized at the same level of theory and basis set. All calculations were performed using “verytight” geometry convergence criteria. No negative harmonic or anharmonic frequencies were observed for all three water hexamer isotopologues indicated above.

## 4 Results and Discussion

### 4.1 Overview of the Experimental Spectra in the 900–4600 cm^–1^ Frequency Range

**Figure 2:**
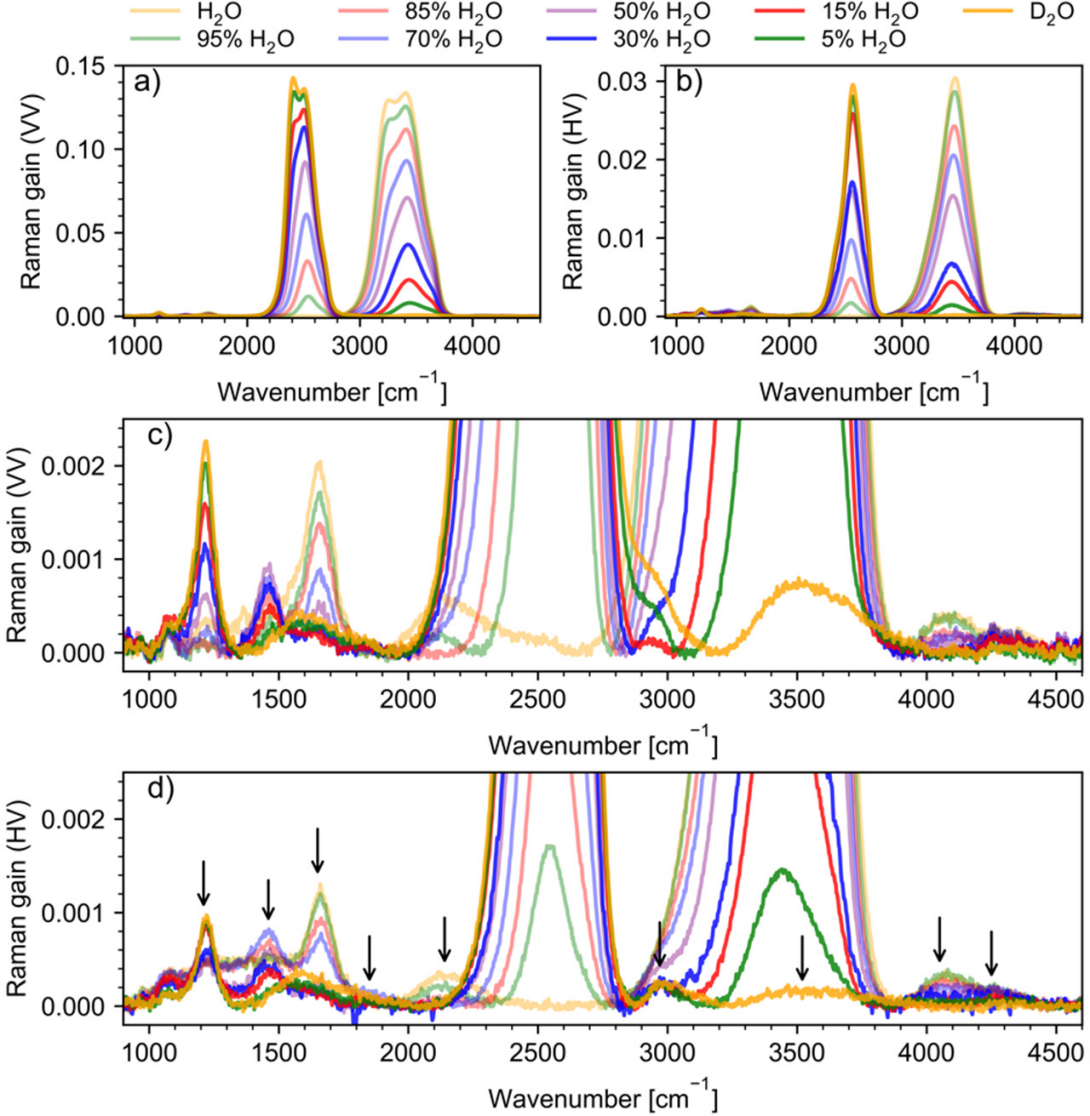
Experimental Raman spectra of H_2_O/D_2_O mixtures, after background subtraction, in the frequency range of 900–4600 cm^–1^ in VV (a) and HV (b) polarizations. Panels (c) and (d) demonstrate experimental VV and HV low-intensity bands. Arrows in panel (d) indicate bands of focus to this work.

Stimulated Raman spectra of H_2_O/D_2_O/HDO mixtures with different concentrations are shown in Figure 2 for VV a) and HV polarization b). Panels c) and d) zoom into low-intensity features, which will be the main focus of this work. We notice that low-intensity bands near 3500 cm^–1^ (dark green) and around 2500 cm^–1^ (light green) in the HV spectra arise from OH and OD stretching vibrations of dilute H_2_O in D_2_O and dilute D_2_O in H_2_O, respectively. We will discuss stretching bands in Sec. 4.2, and in more detail elsewhere, but this work is dedicated to low-intensity features observed in FSRS spectra of H_2_O/D_2_O mixtures.

In what follows, we will discuss low-intensity bands in the 1100–2400 cm^–1^ region that are believed to contain spectroscopic signatures of bending vibrations and combination bands between bending vibrations and librations. We will then discuss low-intensity bands in the 2800–3200 cm^–1^ region that are thought to contain H–O–D bending overtone and OD stretching + librations combination bands. Finally, we will analyze combination bands between OH stretching vibrations and librations, giving rise to low-intensity features in the 3900–4500 cm^–1^ frequency range.

### 4.2 Fundamental OH/OD Bands

**Figure 3:**
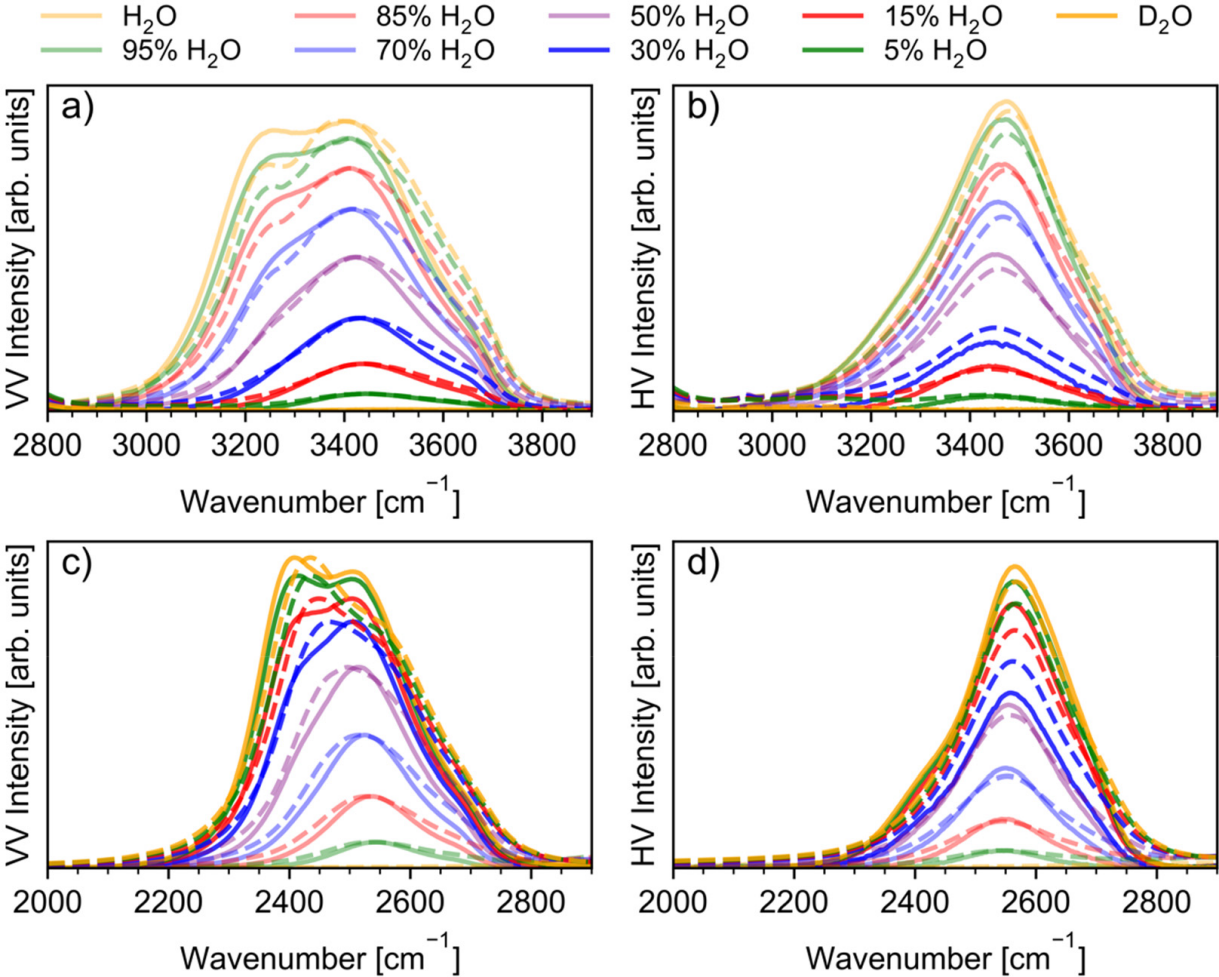
Raman spectra of H_2_O/D_2_O mixtures at 298 K in the OH (a and b) and OD stretching (c and d) frequency ranges. Shown are experimental (solid) and computational (dashed) polarized (VV) and depolarized (HV) spectra. All line shapes were normalized such that experimental and calculated VV spectra have the same peak height, but the relative intensities of the VV and HV peaks were preserved.

We start with a brief discussion of the fundamental OH/OD stretching bands, which dominate spectra of H_2_O/D_2_O mixtures and comprise symmetric ν_1_ and antisymmetric ν_3_ stretching motions. These bands arise from a complex interplay between hydrogen bonding^35,100^ and intra- and intermolecular resonance couplings.^101,102^ They are also influenced by solute–solvent interactions.^103−106^ VV and HV spectra of H_2_O/D_2_O mixtures calculated using the mixed quantum-classical approach described above are compared to the experiment in Figure 3. For each concentration, calculated Raman spectra were normalized such that OH/OD bands in the experimental and calculated VV spectra have the same peak height. However, the relative intensities of the VV and HV peaks were preserved. We find that calculated and experimental spectra are generally in good agreement. Specifically, not only the calculated VV spectra recover the bimodal structure of OH/OD bands observed in the experimental spectra, but they also correctly predict that the high-frequency feature of the bimodal structure in the OH stretch region is more intense than its low-frequency counterpart. The opposite is seen in the relative intensities of the two modes making up the bimodal structure of the VV spectra in the OD stretching range of D_2_O. This has also been correctly reproduced in our simulations. However, in the H_2_O/D_2_O mixtures with concentrations of H_2_O greater than 15%, the relative intensities of the two peaks were not captured. This might be due to an overestimation of the effects of the Fermi resonance on the OD stretching band. Such effects have not been investigated before. It should be noted that the low-frequency feature of the OH stretching region seen in Raman VV spectra appears to be underestimated in the simulated spectra compared to the experiment. The agreement between theory and experiment was better in our previous work which was based on experimental data available at that time.^35^ We experimentally observed that the intensity of the low-frequency feature of the OH band nonlinearly depends on the Raman pulse energy when studied by FSRS. That nonlinearity was observed only in VV polarization, so it is likely related to the polarized “collective” mode of water. We determined the onset of the nonlinear behavior between 0.5 and 0.6 μJ. Therefore, to avoid introducing nonlinear effects into the spectra and to provide the best signal-to-noise ratio of the experiment, the Raman pump was set to 0.5 μJ. Perhaps, that energy still introduced some small nonlinearities causing additional discrepancies between experimental and calculated spectra. Since the presented study was performed with the 515 nm Raman pump, the Raman resonance of the low-frequency part of the OH stretch with the water overtones absorption in the visible red range should not occur.^107^

The discrepancy between the blue sides of the simulated and experimental polarized OH and OD bands has been observed previously and is attributed to the inaccuracy of transition polarizability maps.^35,48^ In light of the new FSRS measurements presented here, further work toward improving transition polarizability maps is in order, and the nonlinear pump dependence of the red side of the OH stretch band will be furthermore examined.

As seen in Figure 3b,c, the relative VV and HV intensities were reproduced well for each concentration. Furthermore, experimental depolarized (HV) bands were reproduced reasonably well in our simulations, especially in the OH stretching range, for almost all concentrations. Both peak positions and full widths at half-maximum (fwhm) are in very good agreement with the experiment.

Since this work is explicitly devoted to the overtones and combinational bands of water, the OD and OH stretching bands will be analyzed in more detail in our future work. Here, it is essential to point out that due to the inhomogeneous broadening of the stretching bands in neat D_2_O and H_2_O, they may cover some low intensity spectral features. One way to better discern low-intensity features is to use isotope diluted water, e.g., HDO in D_2_O. In this case, the OH band is much narrower compared to pure H_2_O.^2^ Moreover, it is known that the low-frequency shoulders of the stretching modes (at around 2400 and 3200 cm^–1^, respectively, for OD and OH stretch) are related to highly polarized collective vibrations of water^108^ and thus are inactive in the HV polarization configuration. Hence, both the isotopic dilution and HV polarization measurements are tools to access low-intensity modes that might be hidden behind the broad OD/OH stretching bands.

### 4.3 Restricted Translational and Librational Modes of the Water Hexamer

In what follows, we attempt to interpret combination bands observed in the experimental Raman spectra in terms of the corresponding modes of the water hexamer. We caution against the direct interpretation of combination bands of liquid water by analogy with those of a finite-size water cluster, but such a comparison can still be very helpful. The optimized geometry of the book hexamer studied in this work can be found in the Supporting Information.

The translational frequency range corresponds to frequencies below 300 cm^–1^. In this range, the calculated Raman VPT2 spectra of the water hexamer are clearly dominated by hydrogen bond bending and stretching. The former involves O–O–O bending with the corresponding feature in the experimental Raman of liquid water found at 62 cm^–1^^13–17.20^ The latter involves hydrogen-bond stretching modes, namely, the O–O stretching along the hydrogen bond direction. In the VPT2 spectra of the water hexamer these modes are found to be in the 94–197 cm^–1^ frequency range. We connect these modes to the broad feature in the experimental Raman spectrum of liquid water with the peak at 175 cm^–1^,^16,20^ which is in good agreement with the calculated frequencies of the book hexamer.

The lowest frequency Raman active librations are associated with a hindered rotation of the water molecule around its *C*_2_ molecular symmetry axis. We label these modes of the hexamer as *v*_c2_. Note that we purposefully use notation that is distinct from *v*_L1_, which was used to label the corresponding band of the liquid water, to emphasize that these modes belong to a cluster. The corresponding frequencies are found to be higher than 210 cm^–1^. We also found that *C*_2_ rotations of some water molecules contribute to librations with frequencies as high as 439 cm^–1^. The corresponding feature in the experimental spectra of liquid water, *v*_L1_, is located at around 450 cm^–1^.^13^ In-plane librations (*v*_ip_) of the water hexamer are found to have frequencies in the 334–754 cm^–1^ range. The corresponding experimental signature, *v*_L2_, is found at around 550 cm^–1^. Finally, out-of-plane wagging librations of the water hexamer, which we denote as *v*_oop_, are found to have frequencies in the range 666–909 cm^–1^, with the corresponding experimental feature, *v*_L3_, centered at around 730 cm^–1^.

We stress that the assignment described above sometimes becomes ambiguous because some modes of the water hexamer involve different motions of different water molecules and, expectedly, have frequencies in between the frequencies of the respective pure modes. In such cases, our assignment is based on the dominating motion. Overall, we find a reasonable correspondence between the librations of the book hexamer and those of liquid water. We will use this to interpret experimentally observed combination bands that involve librations.

### 4.4 The Spectral Range 1100–2400 cm^–1^

**Figure 4:**
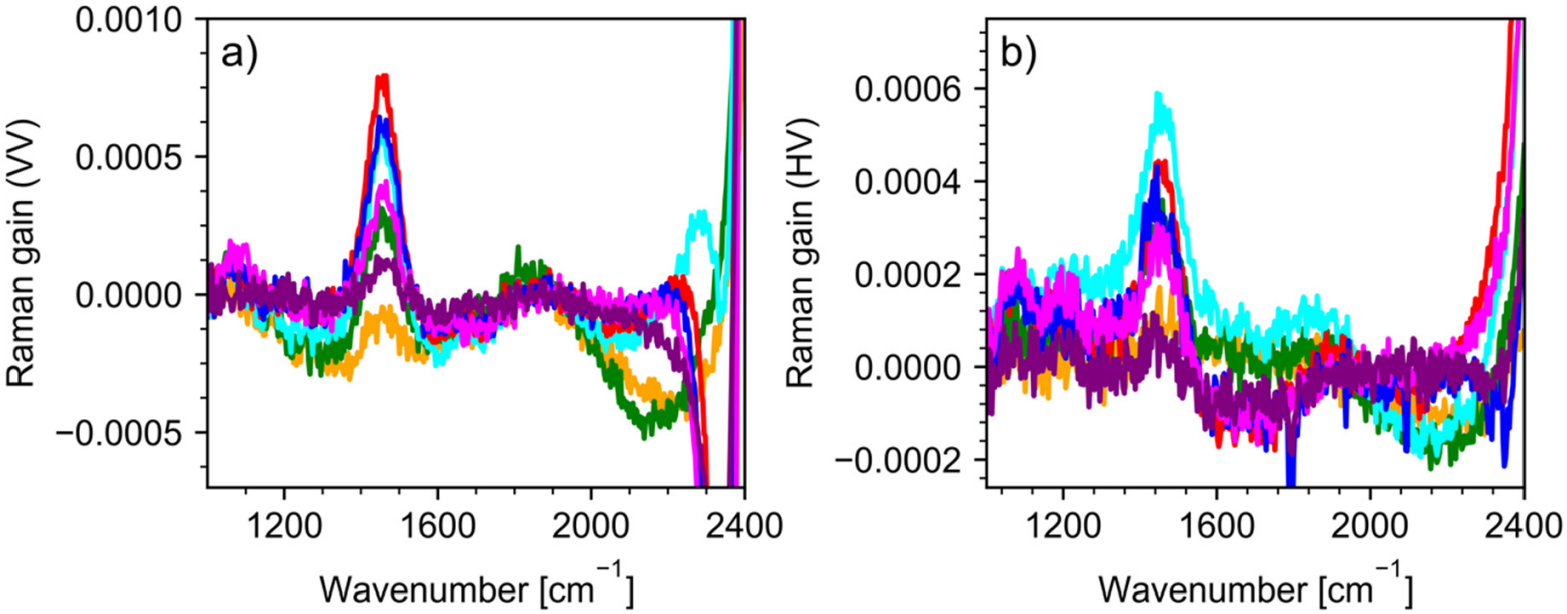
HDO Raman spectra extracted from experimental spectra of H_2_O/D_2_O mixtures by subtracting the spectra of pure components (H_2_O, D_2_O) multiplied by their respected equilibrium fractions calculated from eq 1.

Here we analyze low-intensity features in the 1100–2400 cm^–1^ spectral region of VV and HV Raman spectra of H_2_O/D_2_O mixtures. First, we note that a weak band around 1060–1070 cm^–1^ is associated with silica glass (cuvette). The bending modes, δ, of D_2_O, HDO, and H_2_O are easily identifiable and are located at around 1210, 1460, and 1650 cm^–1^, respectively. Figure 4 illustrates the HDO spectra “extracted” from the spectra of the H_2_O/D_2_O mixtures according to the procedure described in section 2.3. The singled-out HOD bending mode is clearly seen at 1460 cm^–1^. This band has been observed in IR and Raman spectra of liquid water and identified as H–O–D bending before.^109−111^ Moreover, for samples containing up to 15 mol % of H_2_O (for both VV, Figure 2c, and HV, Figure 2d, polarizations), the broad band around 1590 cm^–1^ is seen as well. That band was reported by Walrafen and Blatz as originating from D–O–D bending + libration.^15^ Similarly, we observe a broad band at around 2230 cm^–1^ in the spectra of samples containing ≥95 mol % of H_2_O for VV polarization and ≥85 mol % of H_2_O for HV polarization. In contrast to the combination band at 1590 cm^–1^, the one at 2230 cm^–1^ was reported numerous times and thoroughly analyzed.^15,24,27,112^ It is assigned to H–O–H bending + rocking libration, *v*_L2_.

**Figure 5:**
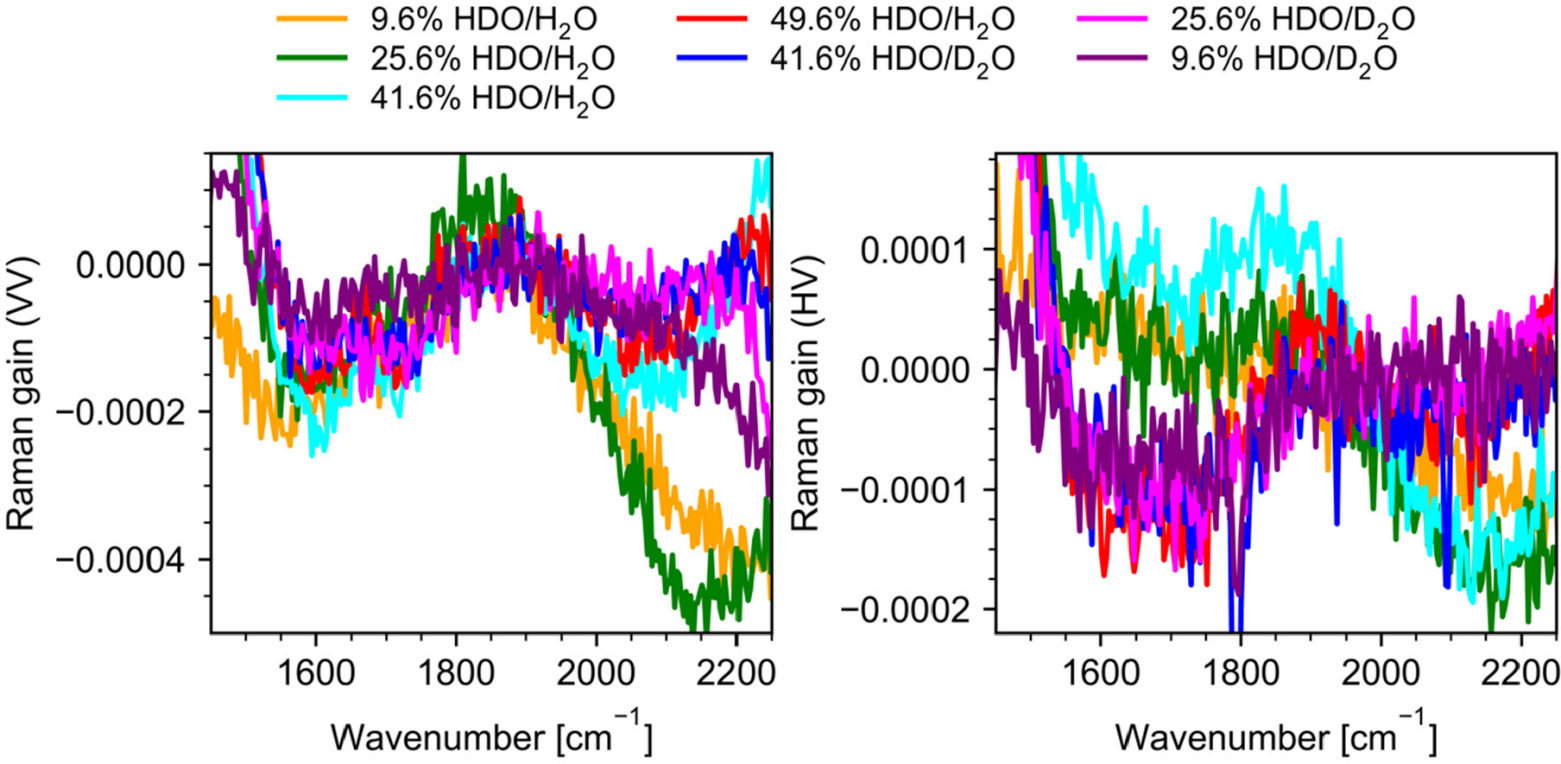
Polarized (VV) and depolarized (HV) Raman spectra of HDO in the 1450–2250 cm^–1^ frequency range obtained from experimental spectra of H_2_O/D_2_O mixtures.

One should also expect the presence of the band related to HOD bending + libration to be located between these two, particularly in the sample containing around 50 mol % of HDO (50/50 H_2_O/D_2_O mixture). Yet, it is not observed in the raw spectra of the mixtures. To uncover this band, we analyze the HDO extracted spectra shown in Figure 5. A peak at 1850 cm^–1^ is clearly seen for both VV and HV polarizations and particularly well for samples containing 49.6 mol % of HDO and 42 mol % of HDO in H_2_O and 25.5 mol % of HDO in H_2_O. We noticed that the peak is most pronounced in the samples in which HDO is “dissolved” in H_2_O, yet presently we could only speculate on the physical background of this fact.

**Figure 6:**
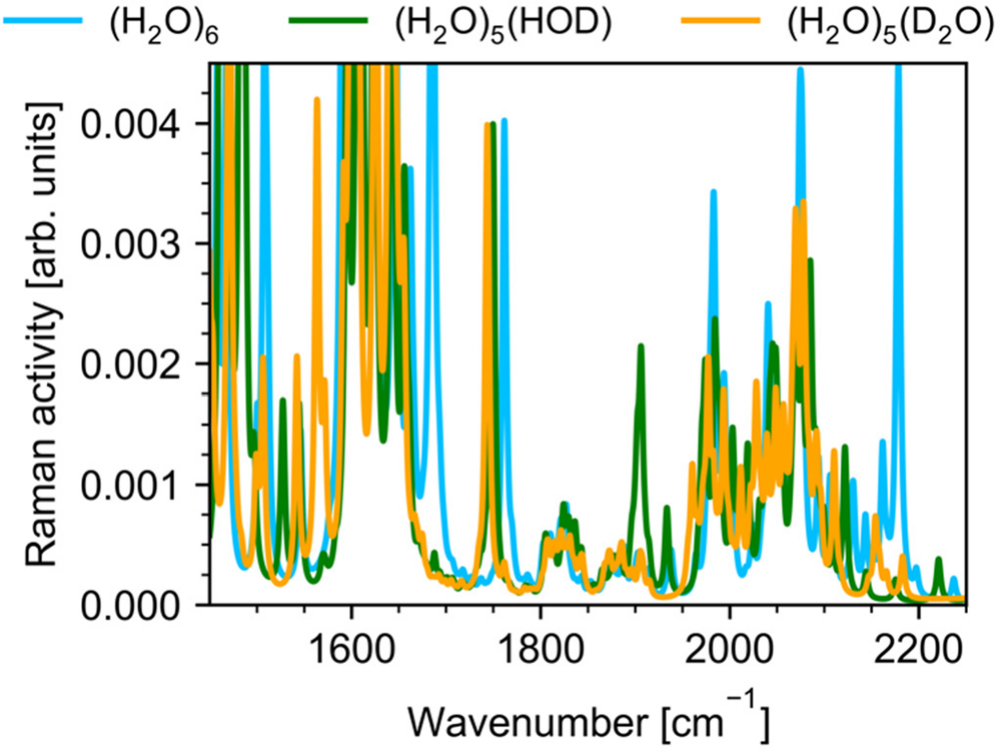
Anharmonic Raman VPT2 spectra of the three isotopologues of the water hexamer in the 1450–2250 cm^–1^ region. Raman spectra of (H_2_O)_5_(HDO) and (H_2_O)_5_(D_2_O) were calculated for the book hexamer in which the water molecule highlighted in Figure 1 was replaced by HDO and D_2_O molecules, respectively. The calculated spectra were convoluted with a 5 cm^–1^ full-width at half-maximum Lorentzian line shape.

Figure 6 shows the anharmonic Raman spectra calculated using vibrational perturbation theory for (H_2_O)_6_, (H_2_O)_5_(HDO), and (H_2_O)_5_(D_2_O) isotopologues of the book hexamer. The calculations were performed at 0 K. For presentation clarity, all peaks were convoluted with a 5 cm^–1^ full-width at half-maximum Lorentzian line shape. Raman spectra of (H_2_O)_5_(HDO), and (H_2_O)_5_(D_2_O) clusters were calculated for the water hexamer with the water molecule highlighted in Figure 1 replaced by HDO and D_2_O molecules, respectively. We choose to alter the isotopic composition of the most strongly hydrogen-bonded water molecule because its bending mode is the most blue-shifted (1686 cm^–1^) compared to the other five anharmonic bending modes at 1645, 1621, 1612, 1606, and 1592 cm^–1^, which makes it easier to identify and track as isotopes are introduced into the structure.

By comparing the Raman spectrum of (H_2_O)_6_ with those of (H_2_O)_5_(HDO) and (H_2_O)_5_(D_2_O), one can follow the spectral changes caused by isotope substitution. First, we note that the combination band designated as bend + libration in the Raman spectra of liquid H_2_O and experimentally observed at around 2140 cm^–1^ (see Figure 2) comprises seven H_2_O bend + libration modes in the computed Raman spectrum of the water hexamer. The corresponding vibrational frequencies were found in the range of 1976–2153 cm^–1^. Close examination of hexamer’s librational modes reveals that the libration modes that contribute to this feature are strongly dominated by in-plane hindered rotations *v*_ip_.

Upon H_2_O to HDO substitution in the book hexamer, the corresponding band shifts to the 1836–1966 cm^–1^ frequency range. The character of the motions that make up this band remains the same: H–O–D bend + *v*_ip_. This result is in good agreement with experimental HDO/H_2_O and HDO/D_2_O Raman spectra that show a band centered at 1850 cm^–1^ (see Figures 4 and 5). Substituting another hydrogen atom of the same water molecule with D further shifts the calculated frequency of now D_2_O bend + libration modes to the 1563–1693 cm^–1^ frequency range where these bands overlap with significantly more intense H–O–H bending modes of the other five water molecules. We note that experimentally D_2_O bend + libration modes give rise to a band centered at 1555 cm^–1^, which is close to the corresponding band in the Raman VPT2 spectra of the water hexamer. Overall, a reasonably good agreement between theory and experiment allows us to confirm that the 2200 cm^–1^ band is indeed a H–O–H bend + *v*_ip_ libration. The frequency shifts to 1850 cm^–1^ in the HDO/H_2_O/D_2_O spectra, but the vibrational character of this band remains the same.

### 4.5 The Spectral Range 2800–3200 cm^–1^

**Figure 7:**
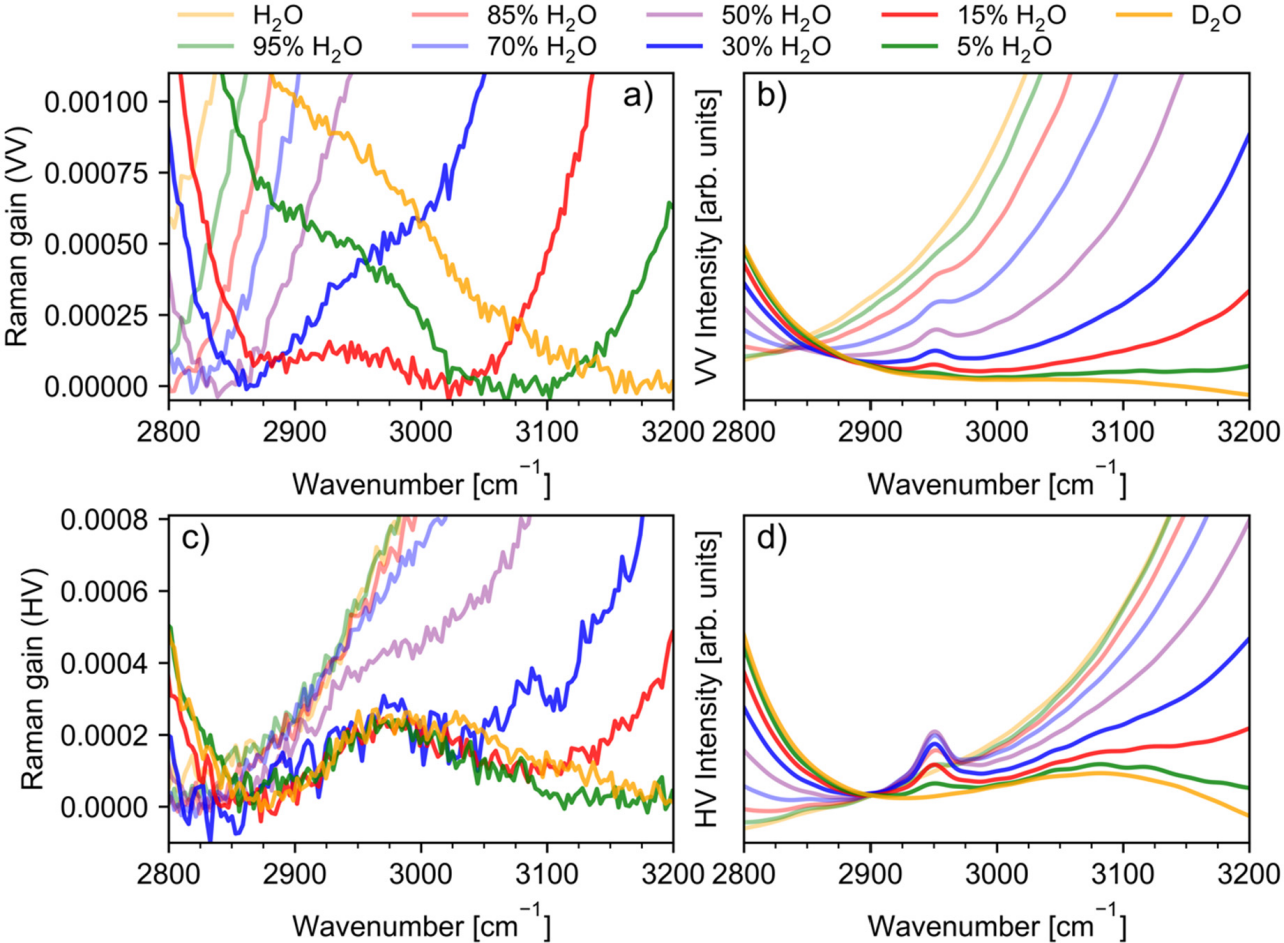
Experimental (a) polarized (VV) and (c) depolarized (HV) and (b) calculated VV and (d) HV Raman spectra in the 2800–3200 cm^–1^ frequency range. Calculated spectra were obtained using mixed quantum-classical approach described in the main text. Note that calculated spectra in this frequency range include HOD bending overtone mode but do not include any combination bands.

**Figure 8:**
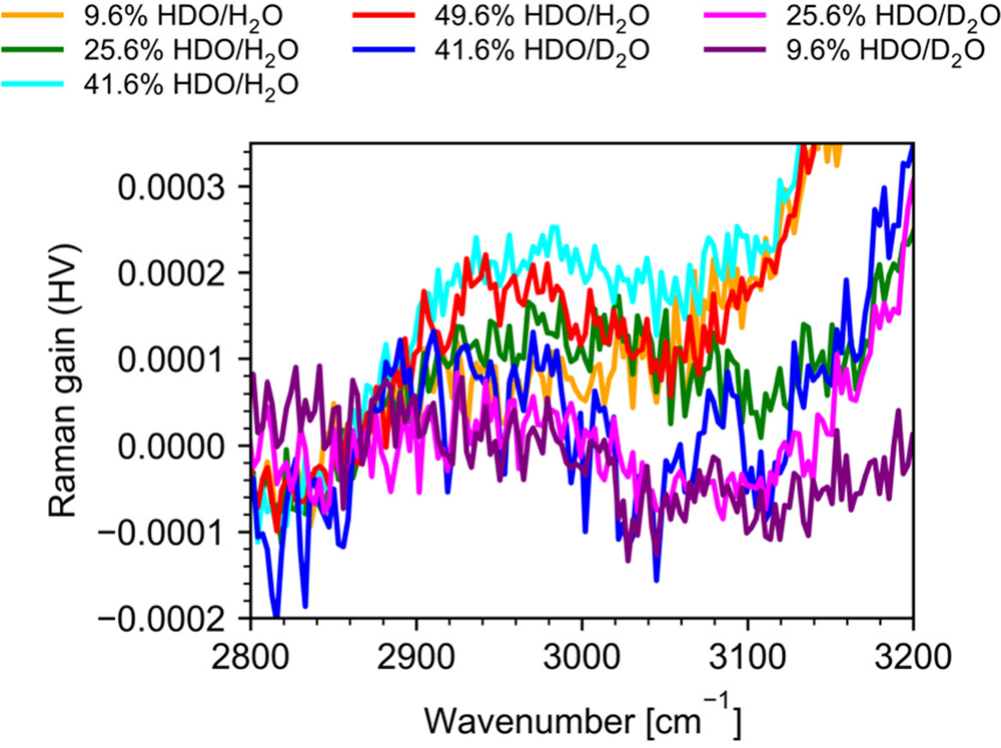
HDO Raman spectra in the HV polarization extracted from the experimental HV Raman spectra in the 2800–3200 cm^–1^ range.

In Figure 7, we show a zoom in into the 2800–3200 cm^–1^ frequency range of the Raman spectra, where a low-intensity spectral feature between 2950 and 3000 cm^–1^ can be observed. In the VV polarized spectrum, this feature is seen just as a shoulder on the blue side of the OD stretching band for the neat D_2_O and mixtures containing 5 mol % of H_2_O and as a red-side shoulder of the OH stretching band in the spectra of mixtures containing more than 15 mol % of H_2_O. This feature is the best separated from both (OD and OH) stretching bands in the sample containing 15 mol % of H_2_O. However, it is seen there just as a small “bump” between intense OD and OH stretching peaks, which causes problems with proper baseline subtraction. As a result, its intensity for the VV polarization is smaller in that range compared to HV polarization. The band at around 2950 cm^–1^ is much better seen in the HV polarized spectra as a distinct band for H_2_O concentrations in the range 0–30 mol %, but it is still discernible in the sample containing 50 mol % of H_2_O. The band’s presence is also evident in HDO “extracted spectra”, containing over 25 mol % of HDO, as illustrated in Figure 8. In pure D_2_O, this band is centered around 2990 cm^–1^ and is somewhat broader compared to the bands in the spectra of H_2_O/D_2_O mixtures, where it is centered around 2975 cm^–1^. A band in this spectral range was observed by Walrafen and Blatz in neat D_2_O (at 2960 cm^–1^) and assigned to the combination band of OD stretch + *v*_L2_.^15^ However, since this mode is also present in the spectra of H_2_O/D_2_O mixtures containing mostly HDO isotopologue, it was assigned by other authors to the overtone of H–O–D bending mode (2δ_HOD_) at 2950 cm^–1^.^59,113^ Since we observe a band in that region in experimental data in neat D_2_O also, we claim, based on our experimental results, that the band around 2970 cm^–1^ contains a contribution from both HDO overtone and a combination band of OD stretch with, most likely, rocking libration *v*_L2_ centered at 425 cm^–1^. The fit of that band with two Gaussian peaks, at 2930 ± 19 and 3020 ± 70 cm^–1^, in the HV spectrum of 50 mol % HDO is shown in Supporting Information, Figure S1.

It is clear from the VV spectra that both modes (2δ_HOD_ and 1ν_OD_ + *v*_L2_) overlap with the red shoulder of the broad OH stretching mode. This overlap is a likely reason for resonant vibrational energy transfer from the OH stretch to the OD stretch in H_2_O/D_2_O/HDO mixtures, possibly through both 2δ_HOD_ and 1ν_OD_ + *v*_L2_, which was observed by de Marco et al. in 2D IR studies^59^ and by Pastorczak et al. in their femtosecond IR pump–SRS probe measurements.^52^

To interpret experimental spectra and assign the observed bands to specific vibrational modes of water, we analyze calculated spectra. First, we discuss VV and HV Raman spectra shown in Figure 7b,d, obtained using a mixed quantum-classical approach. As described above, this approach does not include combination bands. Therefore, while experimental spectra shown in Figure 7a,c show every Raman active mode, the mixed quantum-classical spectra have only H–O–D bending overtone in this frequency range. We reiterate that, in the absence of a spectroscopic map for the HOD bending overtone, its frequency in the excitonic Hamiltonian was set to the experimentally measured value of 2950 cm^–1^.^59^ Moreover, since HOD bending overtone mode remains uncoupled, it is not surprising that this band is not shifted from its uncoupled chromophore frequency 2950 cm^–1^ in the computed spectra of H_2_O/D_2_O mixtures.

Even though our mixed quantum-classical spectra cannot provide insights into combination bands involving librations, we can use them to identify the contribution of the H–O–D bending mode to the band at 2950 cm^–1^. Because there is no H–O–D bending overtone band in the spectra of pure D_2_O, the “difference” between experimental and calculated spectra would show the contribution of the combination bands.

**Figure 9:**
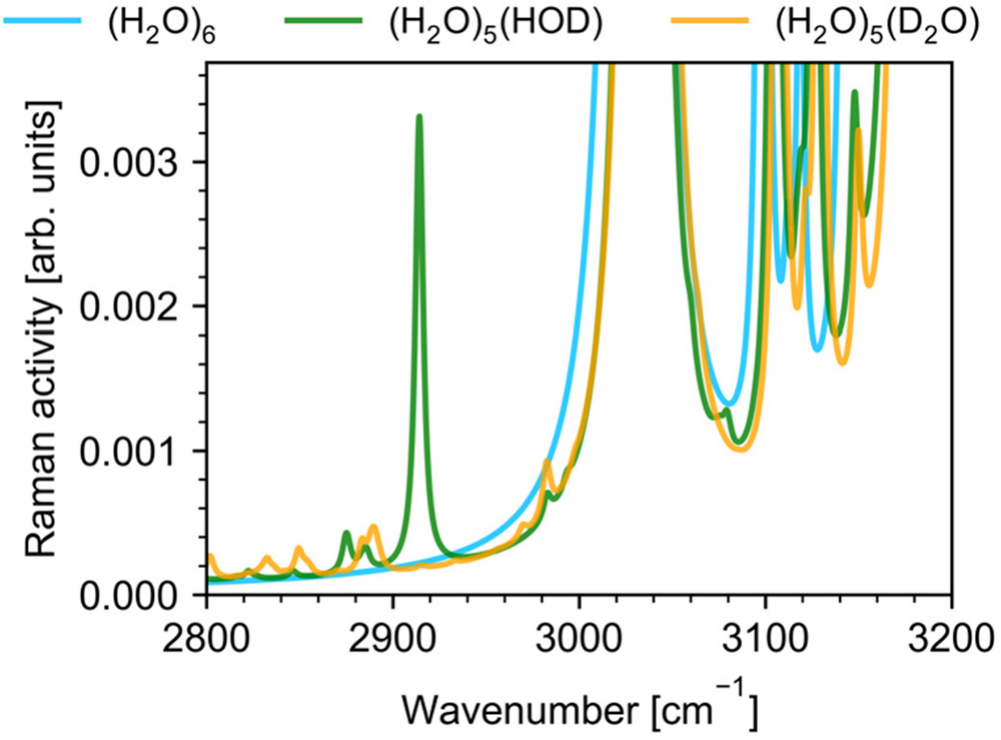
Anharmonic Raman VPT2 spectra of the three isotopologues of the water hexamer in the 2800–3200 cm^–1^ region. (H_2_O)_5_(HDO) and (H_2_O)_5_(D_2_O) spectra are calculated for the hexamer in which the water molecule highlighted in Figure 1 was replaced by HDO and D_2_O molecules, respectively. The calculated spectra were convoluted with a 5 cm^–1^ full-width at half-maximum Lorentzian line shape.

To interpret spectral features in the 2900–3000 cm^–1^ frequency range associated with combination bands, we analyzed VPT2 Raman spectra of the three isotopologues of the water hexamer shown in Figure 9. We observe no spectral features in the calculated Raman spectrum of (H_2_O)_6_ in the 2800–3000 cm^–1^ range in agreement with the experiment. The sharply rising feature at higher frequencies in (H_2_O)_6_ and (H_2_O)_5_(HDO) spectra belongs to the red shoulder of the most red-shifted OH stretching frequency band of the book hexamer located at 3031 cm^–1^.

Calculated anharmonic Raman spectrum of (H_2_O)_5_(HDO) shows a set of combination modes formed by OD stretch fundamental of the HDO molecule (*v*_OD_ = 2611 cm^–1^) and libration modes. There is also H–O–D bending overtone vibration at 2914 cm^–1^ in reasonable agreement with mixed quantum-classical simulations and previous experiments. More combination bands extend beyond 3000 cm^–1^ where they overlap with OH stretching bands. By analyzing the vibrational modes of the book hexamer, we found that lower frequency bands around 2800–2900 cm^–1^ are combination bands involving OD stretching and *v*_*c2*_ librations. The two bands in the spectrum of (H_2_O)_5_(HDO) around 2990 cm^–1^ correspond to OD stretch and in-plane hindered rotations of water molecules *v*_*ip*_.

VPT2 Raman spectrum of (H_2_O)_5_(D_2_O) has more features in the 2900–3000 cm^–1^ frequency range because the cluster has two OD stretches to form combination bands with librations. The high-frequency bands that were observed near 2990 cm^–1^ in (H_2_O)_5_(HDO) spectrum and assigned to OD stretch + *v*_ip_ are present in the (H_2_O)_5_(D_2_O) spectrum at lower frequencies 2850–2870 cm^–1^ with the most intense band having the same character: OD stretch + *v*_ip_. Low-frequency features are shifted to around 2700–2850 cm^–1^ and correspond to lower frequency OD stretch, since there are two OH stretches, mixed with *v*_c2_ (lower range) and *v*_ip_ (higher range) and 2855–3000 cm^–1^ corresponding to higher frequency OD stretch mixed with *v*_c2_ (lower range) and *v*_ip_ (higher range) librations.

Based on our theoretical analysis, we conclude that the band seen in experimental Raman spectra between 2950 and 3000 cm^–1^ has contributions from H–O–D bending overtone as well as from OD stretch + *v*_L2_ (rocking librations) combination band with some contribution from OD stretch + *v*_L1_ (twisting librations) combination band. This assignment fits our experimental results and agrees with the combined assignment of Walrafen and Blatz^15^ and others^59,113^.

### 4.6 The Spectral Range 3200–3900 cm^–1^

Both VV and HV Raman spectra of D_2_O/H_2_O mixtures in the 3200–3900 cm^–1^ frequency range are dominated by the fundamental OH stretching band. OH stretch–H–O–H bend Fermi resonance is believed to be important as well and gives rise to a feature in the VV Raman spectrum at 3250 cm^–1^.^35,58−65^ It is known that the low-frequency feature of the OH stretch decays faster with isotopic dilution than the high-frequency one and the high-frequency feature shifts to higher frequencies. Interestingly, the VV spectrum of pure D_2_O in this region contains a broad peak centered at 3520 cm^–1^. Similarly, the HV spectrum contains a broader feature peaking at roughly the same frequency. This feature is likely due to the contamination of D_2_O with H_2_O. We tried to estimate the “true” level of contamination of D_2_O with H_2_O by looking at the intensity of the H–O–H bending mode. That would either confirm the presence of around 0.4 mol % H_2_O contamination or allow us to extract the spectral residuals unrelated to the contamination. Unfortunately, the H–O–H bending band was weak, barely distinguishable from noise.

**Figure 10:**
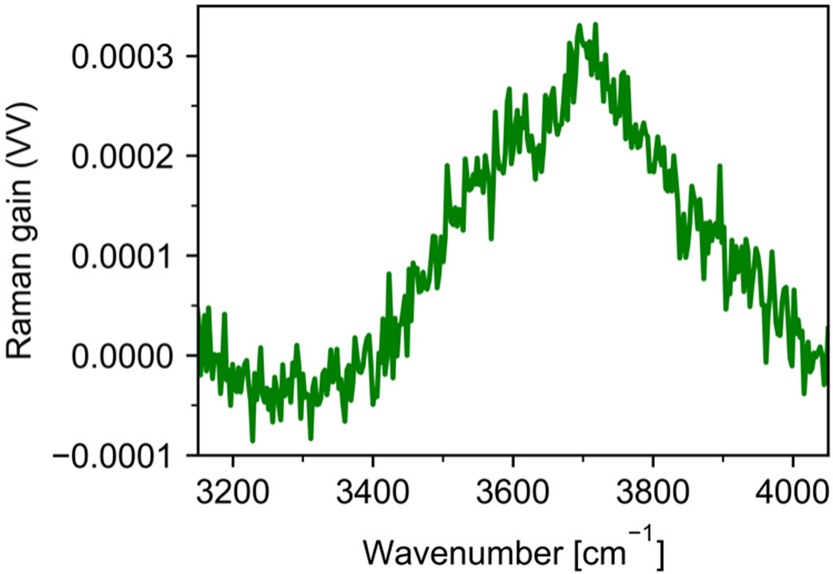
OD stretching + D–O–D bending combination band extracted from experimental Raman VV spectra.

However, H_2_O contamination is not the only contribution to the broad feature observed in the Raman spectrum of D_2_O. The comparison of this band in the D_2_O sample with the OH stretch band in the sample containing 1 mol % of H_2_O shows a clear difference in the central frequency (around 3520 cm^–1^ vs around 3460 cm^–1^) and shapes of respective bands. To illustrate this, we scaled the experimental Raman VV spectra of 1 mol % H_2_O in D_2_O such that its intensity at 3450 cm^–1^ matches that of the spectrum of “pure” D_2_O. The difference between the “pure” D_2_O spectrum and the scaled spectrum of 1 mol % H_2_O is shown in Figure 10. One can clearly identify a peak at 3700 cm^–1^. We assign this difference to a combination band between OD stretching mode (∼2500 cm^–1^) and D–O–D bending mode (∼1210 cm^–1^).^115^

Calculated VPT2 spectra of the water hexamer have two OD stretch + D–O–D bend combination bands located at 3691 and 3837 cm^–1^. The former is in agreement with the experimental result. However, we emphasize that these frequencies are for the particular (H_2_O)_5_(D_2_O) cluster with the D_2_O molecule chosen, as explained above (see Figure 1). Had we designated a different water molecule as a D_2_O molecule, the corresponding frequencies would be different.

### 4.7 The Spectral Range 3900–4500 cm^–1^

Next, we address the weak band located between 4000 and 4100 cm^–1^. It is assumed to originate from the combinational mode OH stretch + libration. The band was initially reported by Walrafen and Blatz^15^ and assigned as a multimode band composed of combinations: OH symmetric stretch (ν_1_) + *v*_L2_ observed at 3990 ± 25 cm^–1^ and OH asymmetric stretch (ν_3_) + *v*_L3_ observed at 4170 ± 50 cm^–1^. Moreover, the authors reported but did not observe experimentally ν_1_ + *v*_L3_ combination mode at 4130 cm^–1^. Walrafen and Puigh, in the later work,^23^ mentioned only the band at 4000 ± 10 cm^–1^ and assigned it to ν_1_ + *v*_L3_ combination mode. Recently, Morawietz et al.^27^ presented measured and *ab initio* MD Raman spectra of liquid water polemizing with the Walrafen’s and Puigh’s interpretation. Their 2D correlation spectra pointed at the correlation of the band observed around 4100 cm^–1^ with the low-frequency libration (*v*_*L1*_) and high-frequency part of the OH stretching band.^27^

**Figure 11:**
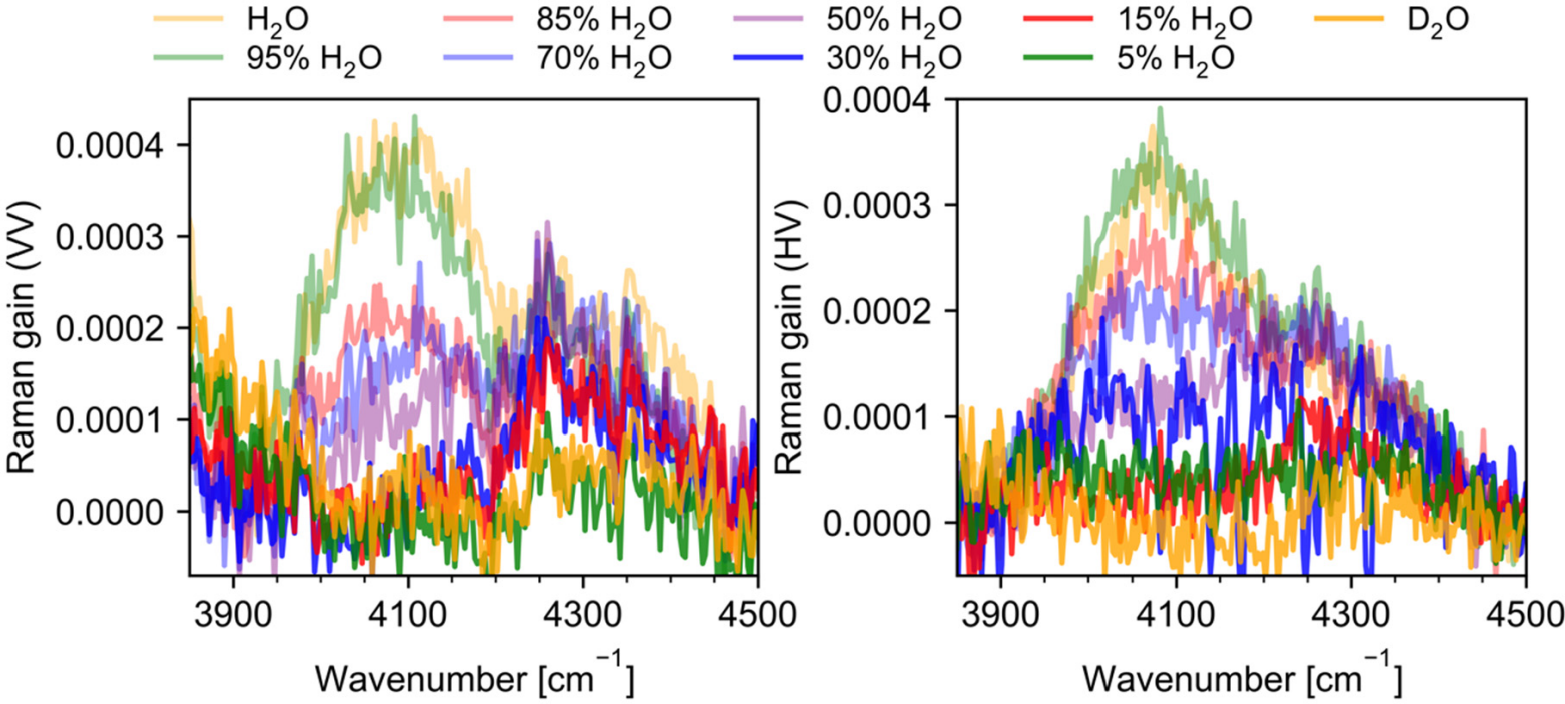
Experimental polarized (VV) and depolarized (HV) Raman spectra of H_2_O/D_2_O mixtures in the 3900–4500 cm^–1^ frequency range.

Our SRS spectra in this frequency range for chosen H_2_O/HDO/D_2_O ratios are shown in Figure 11 for both the VV and HV polarizations. The exact shapes and intensities of bands in this spectral range are difficult to determine for the broad background present therein (see the raw, without baseline subtraction, spectra in Supporting Information, Figure S2), yet at least bimodal structure of the bands is clear. The more intense feature is centered around 4080 cm^–1^, in agreement with the band observed by Morawietz et al., and a weak feature spreads from around 4200 cm^–1^ to about 4460 cm^–1^. A comparison of the intensities of the bands located at 4080 cm^–1^ for both polarization shows that this mode is depolarized.

**Figure 12:**
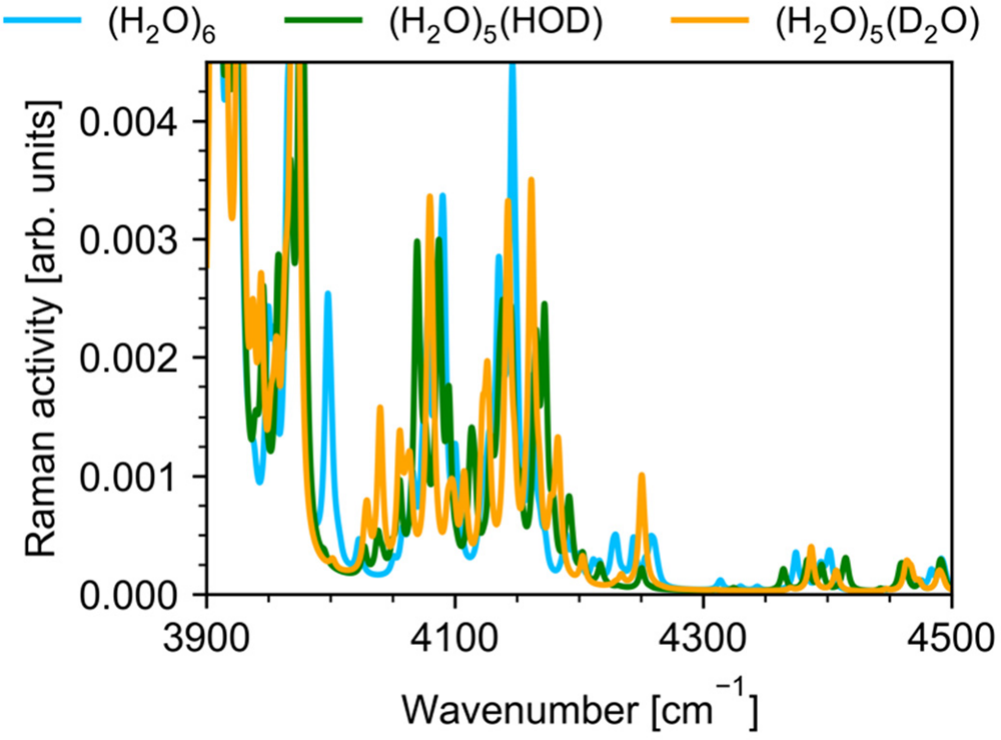
Anharmonic Raman VPT2 spectra of the three isotopologues of the water hexamer in the 3900–4500 cm^–1^ region. (H_2_O)_5_(HDO) and (H_2_O)_5_(D_2_O) spectra are calculated for the hexamer in which the water molecule highlighted in Figure 1 was replaced by HDO and D_2_O molecules, respectively. The calculated spectra convoluted with a 5 cm^–1^ full-width at half-maximum Lorentzian line shape.

To understand spectroscopic features in the 3900–4500 cm^–1^ frequency range, we turn to Raman spectra of water hexamer isotopologues calculated using VPT2 and shown in Figure 12. The spectra of all three isotopologues are similar, for the most part, because the studied water isotopologues differ only by one or two OH groups replaced by OD groups and because all combination bands appearing in this frequency region are OH stretch + libration. Therefore, it suffices to discuss only the VPT2 Raman spectrum of (H_2_O)_6_. The only significant difference between isotopologues spectra is a distinct peak near 4000 cm^–1^ that is present only in the (H_2_O)_6_ spectrum. We find that this peak is a combination band between a *v*_c2_ libration and 3732 cm^–1^ OH stretch whose hydrogen is replaced by D in HDO and D_2_O.

We start our analysis by focusing on the 3950–4200 cm^–1^ frequency range of the VPT2 spectrum of (H_2_O)_6_. We found that the most intense peaks in the lower frequency band 3950–4100 cm^–1^ were clearly dominated by high-frequency OH stretches around 3700 cm^–1^ and *v*_c2_ librations. The higher frequency band 4100–4200 cm^–1^ is found to be dominated by combination modes between high-frequency OH stretches and *v*_ip_ librations.

We also examined VPT2 spectra of the book hexamer for the combination bands formed by lower frequency OH stretching vibrations, 3450 cm^–1^ and below, and librations. We found that combination bands formed by these modes and *v*_oo_ and *v*_ip_ librations appear in the Raman spectrum in 4120–4190 cm^–1^ range but their intensity is much smaller compared to the intensities of combination bands formed by high-frequency OH stretches. Overall, in agreement with Morawietz et al.,^27^ we conclude that combinations bands in this spectral region are dominated by high-frequency OH stretches, but in line with Walrafen and Blatz,^15^ the stretching vibrations are combined with both *v*_L1_ and *v*_L2_ librations.

## Summary and Conclusions

The wavenumbers of the experimentally observed Raman bands for H_2_O, HDO, and D_2_O, together with their assignments supported by our simulations, are summarized in Table 1. Previously, similar analyses regarding overtones and combinational bands were performed by Walrafen and Blatz^15^ for neat H_2_O and D_2_O and later by Walrafen and Pugh^23^ for H_2_O, D_2_O, and HDO in D_2_O (with unspecified concentration). In the former work, the authors postulated many combinational bands involving sums and differences of bending mode with librations: δ + *v*_L1_ – *v*_L3_, δ + *v*_L1_ – *v*_L2_, δ + *v*_L3_ – *v*_L2_ basing on the concavity of a baseline. However, they could not observe ternary sum combinations bands (e.g., δ + v_L1_ + *v*_L3_), which should generally be more intense than the difference bands. Therefore, they called the presence of ternary bands into question. In this work, with the stimulated Raman technique, we were also unable to single out ternary combination bands of water. Nevertheless, we clearly observed combinations of librations with D–O–D bending at 1590 cm^–1^, H–O–H with bending at 2140 cm^–1^, and (for the first time) H–O–D bending at 1850 cm^–1^. For all the isotopologues, the position of the combinational band indicates that the combination cannot involve wagging librations, *v*_L3_, which has the highest energy (565 cm^–1^ for D_2_O and 725 cm^–1^ for H_2_O), so the mixing involves twisting, *v*_L1_ and/or rocking *v*_L2_ librations only. Calculated anharmonic Raman spectra of the water hexamer fully support these conclusions.

**Table 1:** Assignment of Combinational Bands and Some Overtones Observed Experimentally and Calculated (with the VPT2 Method and Mixed Quantum-Classical (QC) Simulations) in the 900–4600 cm^–1^ Range in H_2_O, HDO, and D_2_O

H_2_O (cm^–1^)	HDO (cm^–1^)	D_2_O (cm^–1^)	assignment
1650	1480	1210	bend fundamental (VPT2 and QC^70^)
2140	1850	1590	bend fundamental + rocking libration (VPT2)
	2970		bend overtone (QC, VPT2) and OD stretch + rocking libration (VPT2)
		2970	OD stretch + rocking libration (VPT2)
		3700	OD stretch + D–O–D bend
4050 and 4250	4250		high-frequency OH stretch + rocking libration (VPT2)

To conclude, we measured VV and HV Raman spectra of H_2_O/HDO/D_2_O mixtures of different isotopologue ratios with femtosecond stimulated Raman and compared the results with calculated spectra. Thanks to the superior sensitivity of FSRS and careful measurement procedure, we observed a number of weak low-intensity peaks in the mixture spectra and with the help of computational methods, we assigned them to the combinational modes and overtones of particular isotopologues present in the mixture. Specifically, we observed, for the first time to our knowledge, the mode at around 1850 cm^–1^ and assigned it to H–O–D bend + rocking libration. Second, we found that both H–O–D bend overtone band and OD stretch + rocking libration combination band contribute to the band located between 2850 and 3050 cm^–1^. That mode is most likely responsible for the reported earlier^52,59^ resonant vibrational energy transfer from the OH stretch to OD stretch in the mixture of light and heavy water. Furthermore, we assigned the broad band located between 4000 and 4200 cm^–1^ to be composed of combinational modes of high-frequency OH stretching modes with, predominantly, twisting and rocking librations.

## Supporting Information Available

Additional spectroscopic data with their analyses; detailed description of the mixed quantum-classical simulations of vibrational spectra; Cartesian coordinates of the book hexamer of water used in VPT2 calculations (PDF)
