# Supplementary material for: Combinational Vibration Modes in H2O/HDO/D2O Mixtures Detected Thanks to the Superior Sensitivity of Femtosecond Stimulated Raman Scattering
Source: J Phys Chem B. 2023 May 18;127(21):4843–57. doi: 10.1021/acs.jpcb.3c01334 (PMC10240499; doi:10.1021/acs.jpcb.3c01334)
Supplement: Supplementary file 1 — jp3c01334_si_001.pdf [file jp3c01334_si_001.pdf]

Supporting Information for:

Combinational Vibration Modes in H_2O/HDO/D_2O Mixtures Detected Thanks to the Superior Sensitivity of Femtosecond Stimulated Raman Scattering

Marcin Pastorczak,[1,*] Katsiaryna Duk,[1] Samaneh Shahab,[1] and Alexei A. Kananenka[2,†]

[1]*Institute of Physical Chemistry, Polish Academy of Sciences, Laser Centre, Kasprzaka 44/52, 01-224 Warsaw, Poland*

[2]*Department of Physics and Astronomy, University of Delaware, Newark, DE 19716, USA*

S1. ADDITIONAL EXPERIMENTAL DATA AND ANALYSIS

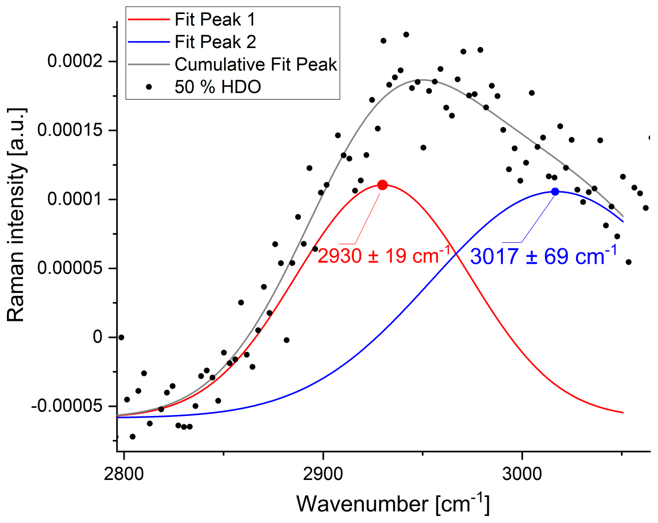

FIG. S1. Example of the fit with two gauss peaks of the band located between 2850 cm^{-1} and 3050 cm^{-1} in the "extracted" 50 mol % of HDO.

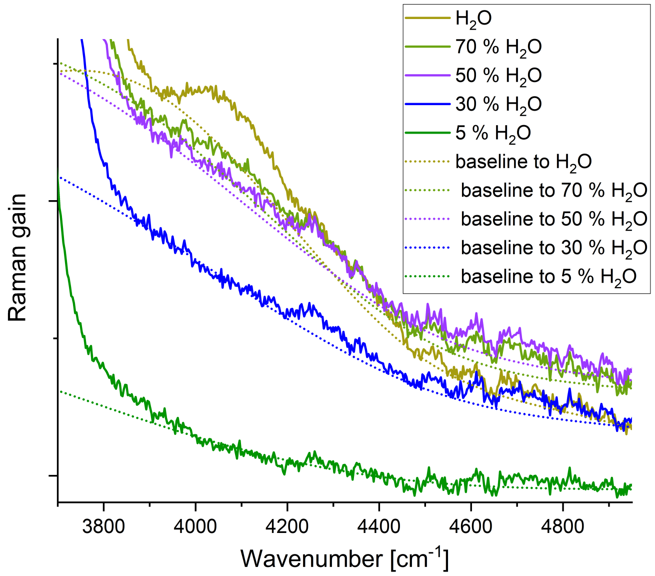

FIG. S2. FSRS spectra of water isotopologues mixture in the 3700-4950 cm^{-1} range without baseline subtraction together with the marked baseline for subtraction. Spectra for only selected concentrations are shown for clarity.

S2. MIXED QUANTUM-CLASSICAL SPECTROSCOPY

Polarized (VV) and depolarized (HV) Raman spectra were calculated by Fourier transform of the corresponding response function $C(t)$ [1]

$$I(\omega) \propto \mathrm{Re} \int_0^\infty dt e^{-i\omega t} C(t), \tag{S1}$$

where $C(t)$ is a time-correlation function of a quantum-mechanical operator $\hat{O}$: $C(t) = \langle \hat{O}(0)\hat{O}(t)\rangle$, where $\hat{O}(t) = e^{i\hat{H}t/\hbar}\hat{O}e^{-i\hat{H}t/\hbar}$ is the Heisenberg representation of the operator $\hat{O}$, $\hat{H}$ is the Hamiltonian of the system, and $\langle \ldots \rangle$ denotes a quantum-mechanical equilibrium statistical average. The particular form of the operator $\hat{O}$ depends on the type of line shape of interest [1]. The following time-correlation functions correspond to VV and HV Raman spectra

$$C_{VV}(t) = C_{iso}(t) + \frac{2}{15} C_{aniso}(t), \tag{S2}$$

$$C_{VH}(t) = \frac{1}{10} C_{aniso}(t), \tag{S3}$$

where $C_{iso}(t)$ and $C_{aniso}(t)$ are isotropic and anisotropic time-correlation functions, correspondingly. [1] They are given by the time-correlation functions of the polarizability tensor $\boldsymbol{\alpha}$. The isotropic Raman response function is the auto-correlation function of the spherical part of the polarizability tensor $\mathcal{A}(t)$ [1]

$$C_{iso}(t) = \langle \mathcal{A}(0)\mathcal{A}(t)\rangle, \tag{S4}$$

where $\mathcal{A}(t) = \frac{1}{3}\mathrm{Tr}[\boldsymbol{\alpha}(t)]$, and $\mathrm{Tr}[\ldots]$ denotes a trace over three-dimensional space. The anisotropic Raman

* mpastorczak@ichf.edu.pl
† akanane@udel.edu

response function is given by the autocorrelation function of the anisotropic polarizability $\mathcal{B}(t)$ [1]

$$C_{aniso}(t) = \langle \text{Tr}\,[\mathcal{B}(0) \cdot \mathcal{B}(t)] \rangle, \tag{S5}$$

where $\mathcal{B}(t) = \alpha(t) - \mathbb{I}\mathcal{A}(t)$, and $\mathbb{I}$ is the unit tensor.

Full quantum-mechanical calculation of Raman line shapes for realistic condensed-matter systems is computationally infeasible due to the prohibitively large size of the Hilbert space required for the description of such systems. Mixed quantum-classical methods often are the only practical ways to calculate Raman spectra for large realistic systems. The basic idea of a mixed quantum-classical approach used in this work is to divide all degrees of freedom in the system into the subsystem comprising vibrations of interest and the so-called environment or "bath", which is composed of slow degrees of freedom [2, 3]. Within this approach vibrations of interest, which are those being probed spectroscopically and typically have frequencies $\hbar\omega >> k_B T$, are treated quantum mechanically (here k_B is the Boltzmann constant). Low-frequency degrees of freedom associated with translations, rotations, and low-frequency vibrations constitute the "bath" and are treated classically. These degrees of freedom ideally (but not necessarily in practice) have frequencies less than $k_B T$. Finally, there are other degrees of freedom, typically high-frequency vibrations that are thought not to contribute to the spectroscopy of interest, and therefore are ignored. All bath degrees of freedom are treated classically via classical molecular dynamics. High-frequency OH (OD) stretch and HOH (DOD, HDO) bend vibrations are treated quantum-mechanically as explained below.

We used the one-exciton model to describe molecular vibrations (chromophores). In the local-mode basis the vibrational exciton Hamiltonian is given by

$$H = H_0 |0\rangle\langle 0| + \sum_{nm} H_{nm} |n\rangle\langle m|. \tag{S6}$$

This Hamiltonian describes a system with the ground state $|0\rangle$ where all chromophores are in their ground states with H_0 being the corresponding Hamiltonian. The system is also composed of a manifold of N singly excited states, $\{|n\rangle : n = 1, \ldots, N\}$, corresponding to a single excitation on the nth chromophore with all other chromophores in their ground states: $|n\rangle = |0_1\rangle \ldots |1_n\rangle \ldots |0_N\rangle$. For $n = m$ the Hamiltonian H_{nm} describes the local excitation on chromophore n; otherwise it is the coupling between n and m chromphores.

Within the mixed quantum-classical theory based on the one-exciton model above, for a system of coupled chromophores Raman line shape can be written as [4]

$$I(\omega) \propto \text{Re} \int_0^\infty dt e^{-i\omega t} \tilde{C}(t). \tag{S7}$$

where $\tilde{C}(t)$ is the corresponding (e.g., VV or HV) mixed quantum-classical time-correlation functions [1]

$$\begin{aligned} \tilde{C}_{VV}(t) &= \frac{1}{15} \sum_{jkpq} \langle a_{jpp}(0) F_{jk}(t) a_{kqq}(t) \\ &+ 2 a_{jpq}(0) F_{jk}(t) a_{kpq}(t) \rangle e^{-t/2T_1}, \end{aligned} \tag{S8}$$

$$\begin{aligned} \tilde{C}_{VH}(t) &= \frac{1}{30} \sum_{jkpq} \langle 3 a_{jpq}(0) F_{jk}(t) a_{kpq}(t) \\ &- a_{jpp}(0) F_{jk}(t) a_{kqq}(t) \rangle e^{-t/2T_1}, \end{aligned} \tag{S9}$$

where a_{jpq} is the $p, q \in \{x, y, z\}$ component of the transition polarizability tensor for chromophore j, $\hat{p}$ is the unit vector in the direction of polarization of the incident light beam, $\hat{q}$ is the unit vector in the direction of polarization of the scattered beam, T_1 is the average lifetime of the vibrationally excited states which is taken to be 260 ps [5]. $F_{jk}(t)$ are the matrix elements of matrix $\mathbf{F}(t)$, which satisfies the following equation of motion

$$\frac{d\mathbf{F}(t)}{dt} = i\mathbf{F}(t)\mathbf{K}(t), \tag{S10}$$

where the matrix elements of $\mathbf{K}(t)$ are given by

$$K_{nm}(t) = \omega_n(t)\delta_{nm} + \omega_{nm}(t)\left(1 - \delta_{nm}\right), \tag{S11}$$

where the site frequencies are denoted by ω_n, and $\{\omega_{nm} : n \neq m\}$ are the vibrational chromophore-chromophore couplings. If n and m belong to two chromophores on the same molecule, then ω_{nm} is the intramolecular coupling element and will be denoted by $\omega_{nm}^{\text{intra}}$, while if n and m belong to different molecules, ω_{nm} is an intermolecular coupling element and will be denoted as $\omega_{nm}^{\text{inter}}$. Site frequencies as well as inter- and intramolecular couplings generally change with time due to fluctuations of the bath variables, giving rise to time-dependence of the matrix $\mathbf{K}(t)$.

Numerical solution of Eq. (S10), subject to the initial condition $F_{nm}(0) = \delta_{nm}$, produces a set of time-evolved matrices $\mathbf{F}(t)$, which are used to calculate Raman line shapes according to Eqs. (S8)–(S9).

In addition to the time-propagation matrix $\mathbf{F}(t)$, knowledge of the components of the time-evolved transition polarizability tensor $\boldsymbol{\alpha}$ for each chromophore is required in order to calculate Raman line shapes.

Matrix elements of the transition polarizability are approximated using the bond-polarizability model as follows [6]

$$a_{pq} = \left(\alpha'_{\parallel} - \alpha'_{\perp}\right) x\hat{p} \cdot \hat{u}\; \hat{u} \cdot \hat{q} + \alpha'_{\perp} x\hat{p} \cdot \hat{q}, \tag{S12}$$

where $\alpha'_{\parallel}$ ($\alpha'_{\perp}$) are the longitudial (transverse) bond polarizability derivatives. The simulated spectra are determined by the ratio $\alpha'_{\parallel}/\alpha'_{\perp}$. In this work we use $\alpha'_{\parallel}/\alpha'_{\perp} = 5.6$ as experimentally obtained by Scherer and Snyder [7].

Intramolecular and intermolecular chromophore-chromophore couplings are also required to construct

matrix **K**. The intramolecular coupling between two OH (OD) stretch chromophores n and m is given by [8, 9]

$$\hbar\omega_{nm}^{\mathrm{intra}} = k_{nm}^{\mathrm{intra}} x_n x_m + \frac{\cos\phi}{m_{\mathrm{O}}} p_n p_m, \qquad \text{(S13)}$$

where p_n is the 1–0 momentum matrix elements of OH (OD) stretching chromophore n, ϕ is the angle between OH (OD) stretch chromophores n and m, which is fixed at 104.52° for the rigid water model used in this work, m_{O} is the mass of oxygen atom and k_{nm}^{intra} is the second derivative of the potential energy with respect to O–H(D) bond lengths, calculated at r_{OH}^{eq}. Due to frequency mismatch intramolecular couplings in HOD molecules were set to 0.

The intermolecular coupling between two OH (OD) stretch chromophores n and m is described using transition dipole coupling (TDC) scheme

$$\hbar\omega_{nm}^{\mathrm{inter}} = k_{nm}^{\mathrm{inter}} x_n x_m, \qquad \text{(S14)}$$

where k_{nm}^{inter} is given by

$$k_{nm}^{\mathrm{inter}} = \frac{\mu_n'\mu_m'\{\hat{u}_n\cdot\hat{u}_m - 3\left[(\hat{u}_n\cdot\hat{n}_{nm})(\hat{u}_m\cdot\hat{n}_{nm})\right]\}}{r_{nm}^3}, \qquad \text{(S15)}$$

where $\hat{n}_{nm}$ is a unit vector along the line connecting the locations of OH (OD) stretch transition dipoles n and m, and r_{nm} is the distance between these locations. The transition dipole of an OH (OD) stretch chromophore is located along the OH (OD) bond 0.67 Å away from the oxygen atom [9].

The nature of the intermolecular coupling between bend fundamental vibrations is less clear [10, 11]. However, because in this work we are only concerned with the bend overtone vibration and, since its 2–0 transition matrix element is smaller than the 1–0 transition matrix element, the intermolecular coupling between bend overtone modes is neglected.

The intramolecular coupling between the fundamental OH stretching and overtone of the HOH bending vibrations is known as Fermi resonance coupling. As in the previous work it is assumed constant [12, 13]. Its value 25 cm^{-1} (in the local-mode basis) is determined by fitting the calculated polarized Raman spectra to its experimental counterpart [12]. This value is in a good agreement with earlier works based on somewhat similar fitting strategies [14–18]. Fermi resonance in D_2O and HDO was ignored because, in contrast to H_2O, the value of the Fermi resonance coupling in such systems has not been determined.

It remains to describe how transition frequencies ω_n, vibrational couplings ω_{nm}, dipole moment derivatives μ_n', coordinate x_n, and momentum p_n matrix elements change with time. As done previously [3, 19, 21, 22], we correlate vibrational frequencies with the effective electric field computed for each configuration of water molecules at each time step from the point charges utilized in the molecular dynamics force field. The OH (OD) stretch frequency is determined from the electric field E at the location of the hydrogen (deuterium) atom along the direction of the OH (OD) bond. The mapping relationships between E and OH (OD) stretch transition frequencies ω_n are determined so as to reproduce the correct distribution of vibrational OH (OD) stretch frequencies determined by the density functional theory calculations.

For example, in order to calculate transition frequencies and dipole derivatives of the OH-stretch, a MD trajectory with E3B2 force field was generated first. Then clusters of water molecules centered on a randomly chosen water hydrogen atom were selected from the MD trajectory and electronic structure calculations are performed to determine transition frequency and transition dipole moment of the OH stretch of the central molecule [19]. All water molecules whose oxygen atom was within a cutoff of 4.0 Å of the central hydrogen atom were explicitly included in the calculation, while all waters between 4.0 and 7.831 Å were implicitly in-

TABLE S1. Spectroscopic maps used in this work. See Refs. 9, 19, and 20 for details on OH and OD stretching maps and Ref. 10 for details of HOH and DOD bending maps. E is the effective electric field at the location of the water H (D) atom along the direction of OH (OD) bond and $\mathcal{E}$ denotes the sum of the components of electric fields perpendicular to the two OH (OD) bonds and pointing towards the HOH (DOD) bisector.

Quantity	Units
OH stretch fundamental:	
$\omega_{\mathrm{OH}} = 3760.2 - 3541.7E - 152677E^2$	[cm^{-1}]
$x_{\mathrm{OH}} = 0.19285 - 1.7261\times10^{-5}\omega_{\mathrm{OH}}$	[a.u.]
$p_{\mathrm{OH}} = 1.6466 + 5.7692\times10^{-4}\omega_{\mathrm{OH}}$	[a.u.]
OD stretch fundamental:	
$\omega_{\mathrm{OD}} = 2767.8 - 2630.3E - 102601E^2$	[cm^{-1}]
$x_{\mathrm{OD}} = 0.16593 - 2.0632\times10^{-5}\omega_{\mathrm{OD}}$	[a.u.]
$p_{\mathrm{OD}} = 2.0475 + 8.9108\times10^{-4}\omega_{\mathrm{OD}}$	[a.u.]
Both OH and OD stretch fundamental:	
$\mu' = 0.1646 + 11.39E + 63.41E^2$	[a.u.]
$\omega_{nm}^{\mathrm{intra}} = [-1361 + 27165(E_n + E_m)]x_n x_m - 1.887 p_n p_m$	[a.u.]
$\alpha_{\parallel}'/\alpha_{\perp}' = 5.6$	
HOH bend overtone:	
$\omega_{\mathrm{HOH}} = 3132.78 + 6086.31\mathcal{E}$	[cm^{-1}]
$\omega^{\mathrm{inter}} = 0$	[cm^{-1}]
DOD bend overtone:	
$\omega_{\mathrm{HOH}} = 2380$	[cm^{-1}]
$\omega^{\mathrm{inter}} = 0$	[cm^{-1}]
HOD bend overtone:	
$\omega_{\mathrm{HOH}} = 2950$	[cm^{-1}]
$\omega^{\mathrm{inter}} = 0$	[cm^{-1}]
Fermi resonance coupling:	
$\omega_{\mathrm{HOH+OH}}^{\mathrm{intra}} = 25$	[cm^{-1}]
$\omega_{\mathrm{DOD+OD}}^{\mathrm{intra}} = 0$	[cm^{-1}]
$\omega_{\mathrm{HOD+OH/OD}}^{\mathrm{intra}} = 0$	[cm^{-1}]

cluded only as point charges according to E3B2 water model (+0.52e for hydrogen and -1.04e for the dummy atom M site [23]). The OH bond of the central water molecule was systematically stretched and contracted to map out a one-dimensional potential energy surface using the Gaussian software package [24]. The selected OH bond is varied between 0.6572 Å and 1.7572 Å with a grid spacing of 0.02 Å.The coordinates of all other atoms in the cluster are kept fixed as the bond length is changed. At each bond length, both the total energy and total dipole moment of the system are calculated at the B3LYP/6-311++G(d,p) level of theory and basis set [25–27] using the GAUSSIAN09 quantum chemistry program. It should be noted that the same procedure performed for the HOD monomer leads to OH-stretch frequency of 3699 cm^{-1} [19] which can be compared to the experimental value of 3707 cm^{-1} [28]. To correct for this small difference, we multiply all calculated transition frequencies by the scale factor of 1.0021. Similar approach was also used to determine OD stretching frequency.

The OH and OD dipole derivative μ' map is determined similarly as a function of E by a quadratic least-squares fit to the data from the B3LYP/6-311++G** calculations. Coordinate x and momentum p matrix elements were found to depend linearly on the transition frequency. The intramolecular coupling $\omega_{nm}^{\text{intra}}$ was fit to a linear function of the sum of the electric fields for chromophores n and m.

Both the HOH bend fundamental transition frequency and first overtone frequency are correlated with the sum of the components of electric fields perpendicular to the two OH bonds and pointing towards the HOH bisector, evaluated at the hydrogen atoms [10]. HOH bending maps for 1–0 and 2–1 transitions were determined previously [10]. Since in this work the 2–0 transition (first overtone) frequency is needed, it was obtained as the sum of 1–0 and 2–1 transition frequencies from Ref. 10. Note that the 2–1 frequency map includes an anharmonicity of ~30 cm^{-1}. Because DOD and HOD bending maps have not been developed, in this work, we set their overtone frequencies to 2380 and 2950 cm^{-1} correspondingly as estimated from experimental spectra [29, 30].

A summary of all spectroscopic maps and other parameters used in this work is illustrated in Table S1.

S3. BOOK HEXAMER GEOMETRY

Cartesian coordinates of all atoms of the water hexamer used in VPT2 calculations are shown in Tab. S2.

TABLE S2. Cartesian coordinates of the book isomer of the water hexamer optimized using B3LYP/6-311++G(d,p) level of theory and basis set.

Atom	X [Å]	Y [Å]	Z [Å]
O	1.568608	-0.122695	0.827234
H	1.531067	-0.973684	0.321321
H	2.156409	-0.272852	1.574047
O	1.194077	-2.443979	-0.483844
H	0.208963	-2.529319	-0.496215
H	1.493707	-2.628495	-1.378514
O	-1.497309	-2.318274	-0.405404
H	-2.085903	-2.929704	0.045878
H	-1.600522	-1.443528	0.046457
O	-1.379926	2.517089	-0.383945
H	-0.408334	2.591536	-0.484093
H	-1.670095	3.334442	0.030775
O	1.385815	2.345059	-0.457347
H	1.561961	1.476416	-0.041780
H	1.915104	2.374263	-1.259326
O	-1.383549	0.031358	0.909984
H	-0.435459	0.061965	1.114580
H	-1.562728	0.883421	0.463899

[1] D. A. McQuarrie, *Statistical Mechanics* (University Science Books, California, 2000).
[2] J. L. Skinner, B. M. Auer, and Y.-S. Lin, Adv. Chem. Phys. **142**, 59 (2009).
[3] B. M. Auer and J. L. Skinner, J. Chem. Phys. **128**, 224511 (2008).
[4] B. M. Auer and J. L. Skinner, J. Chem. Phys. **129**, 214705 (2008).
[5] A. J. Lock and H. J. Bakker, J. Chem. Phys. **117**, 1708 (2002).
[6] A. Belch and S. Rice, J. Chem. Phys. **78**, 4817 (1983).
[7] J. G. Scherer and R. G. Snyder, J. Chem. Phys. **67**, 4794 (1977).
[8] E. B. Wilson, J. C. Decius, and P. C. Cross, *Molecular Vibrations* (Dover, New York, 1980).
[9] F. Li and J. L. Skinner, J. Chem. Phys. **133**, 244504 (2010).
[10] Y. Ni and J. L. Skinner, J. Chem. Phys. **143**, 014502 (2015).
[11] Y. Wang, X. Huang, B. C. Shepler, B. J. Braams, and J. M. Bowman, J. Chem. Phys. **134**, 094509 (2011).
[12] A. A. Kananenka and J. L. Skinner, J. Chem. Phys. **148**, 244107 (2018).
[13] A. A. Kananenka, N. J. Hestand, and J. L. Skinner, J. Phys. Chem. B **123**, 5139 (2019).
[14] D. F. Smith Jr. and J. Overend, Spectrochim. Acta Part A **28**, 471 (1972).
[15] J. R. Scherer, M. K. Go, and S. Kint, J. Phys. Chem. **78**, 1304 (1974).
[16] J. E. Baggott, Mol. Phys. **65**, 739 (1988).
[17] H. G. Kjaergaard, B. R. Henry, H. Wei, S. Lefebvre, T. Carrington, O. S. Mortensen, and M. L. Sage, J. Chem. Phys. **100**, 6228 (1994).
[18] A. B. McCoy and E. L. Silbert III, J. Chem. Phys. **92**, 1893 (1990).
[19] S. M. Gruenbaum, C. J. Tainter, L. Shi, Y. Ni, and J. L. Skinner, J. Chem. Theory Comput. **9**, 3109 (2013).

[20] L. Shi, S. M. Gruenbaum, and J. L. Skinner, J. Phys. Chem. B **116**, 13821 (2012).

[21] B. M. Auer, R. Kumar, J. R. Schmidt, and J. L. Skinner, Proc. Natl. Acad. Sci. USA **104**, 14215 (2007).

[22] F. Li and J. L. Skinner, J. Chem. Phys. **132**, 204505 (2010).

[23] W. L. Jorgensen, J. Chandrasekhar, J. D. Madura, R. W. Impey, and M. L. Klein, J. Chem. Phys. **79**, 926 (1983).

[24] M. J. Frisch, G. W. Trucks, H. B. Schlegel, G. E. Scuseria, M. A. Robb, J. R. Cheeseman, G. Scalmani, V. Barone, B. Mennucci, G. A. Petersson, H. Nakatsuji, M. Caricato, X. Li, H. P. Hratchian, A. F. Izmaylov, J. Bloino, G. Zheng, J. L. Sonnenberg, M. Hada, M. Ehara, K. Toyota, R. Fukuda, J. Hasegawa, M. Ishida, T. Nakajima, Y. Honda, O. Kitao, H. Nakai, T. Vreven, J. J. A. Montgomery, J. E. Peralta, F. Ogliaro, M. Bearpark, J. J. Heyd, E. Brothers, K. N. Kudin, V. N. Staroverov, R. Kobayashi, J. Normand, K. Raghavachari, A. Rendell, J. C. Burant, S. S. Iyengar, J. Tomasi, M. Cossi, N. Rega, J. M. Millam, M. Klene, J. E. Knox, J. B. Cross, V. Bakken, C. Adamo, J. Jaramillo, R. Gomperts, R. E. Stratmann, O. Yazyev, A. J. Austin, R. Cammi, C. Pomelli, J. W. Ochterski, R. L. Martin, K. Morokuma, V. G. Zakrzewski, G. A. Voth, P. Salvador, J. J. Dannenberg, S. Dapprich, A. D. Daniels, Ö. Farkas, J. B. Foresman, J. V. Ortiz, J. Cioslowski, and D. J. Fox, "Gaussian 09, Revision B.01," Gaussian Inc., Wallingford CT (2009).

[25] A. D. Becke, J. Chem. Phys. **98**, 5648 (1993).

[26] C. Lee, W. Yang, and R. G. Parr, Phys. Rev. B **37**, 785 (1988).

[27] R. Krishnan, J. S. Binkley, R. Seeger, and J. A. Pople, J. Chem. Phys. **72**, 650 (1980).

[28] W. S. Benedict, N. Gailar, and E. K. Plyler, J. Chem. Phys. **24**, 1139 (1956).

[29] L. De Marco, K. Ramasesha, and A. Tokmakoff, J. Phys. Chem. B **117**, 15319 (2013).

[30] M. Falk, J. Raman Spectrosc. **21**, 563 (1990).
